# Interpretable Multiscale Convolutional Neural Network for Classification and Feature Visualization of Weak Raman Spectra of Biomolecules at Cell Membranes

**DOI:** 10.1021/acssensors.4c03260

**Published:** 2025-04-04

**Authors:** Che-Lun Chin, Chia-En Chang, Ling Chao

**Affiliations:** Department of Chemical Engineering, National Taiwan University, No. 1, Sec. 4, Roosevelt Rd., Taipei 10617, Taiwan

**Keywords:** convolutional neural networks (CNN), Raman spectroscopy, interpretable, multiscale, biomolecular spectra

## Abstract

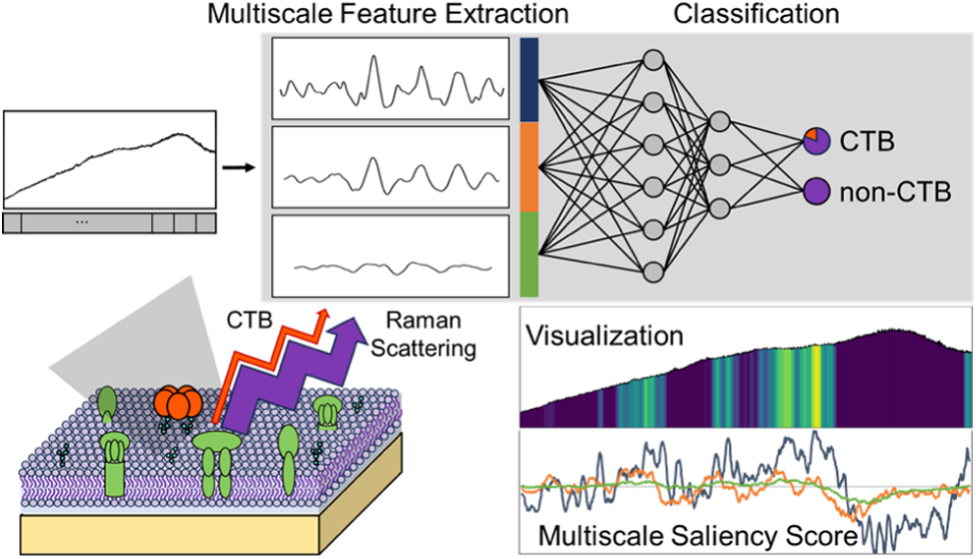

Raman spectroscopy in biological applications faces challenges due to complex spectra, characterized by peaks of varying widths and significant biological background noise. Convolutional neural networks (CNNs) are widely used for spectrum classification due to their ability to capture local peak features. In this study, we introduce a multiscale CNN designed to detect weak biomolecule signals and differentiate spectra with features that cannot be statistically distinguished. The approach is further enhanced by a new visualization technique tailored for multiscale spectral analysis, providing clear insights into classification results. Using the classification of cholera toxin B subunit (CTB)-treated versus untreated cell membrane samples, whose spectra cannot be statistically differentiated, the optimized multiscale CNN achieved superior performance compared to traditional machine learning methods and existing multiscale CNNs, with accuracy (99.22%), sensitivity (99.27%), specificity (99.16%), and precision (99.20%). Our new visualization method, based on gradients of activation maps with respect to class scores, generates saliency scores that capture sample variations, with decision-making relying on consistently identified peak features. By visualizing the effects of different kernel sizes, Grad-AM highlights features at varying scales, aligning closely with spectral features and enhancing CNN interpretability in complex biomolecular analysis. These advancements demonstrate the potential of our method to improve spectral analysis and reveal previously hidden peaks in complex biological environments.

Raman spectroscopy is well-suited for studying biomolecules due to its nondestructive nature, minimal sample preparation, and compatibility with aqueous environments. It generates unique vibrational fingerprints, providing chemical identity and enabling label-free detection.^1,2^ This technique has been extensively applied in biosensing^3−5^ and biomedical research.^6−8^ However, interpreting Raman spectra of biomolecules at low concentrations in complex biological systems remains challenging, as the signals are often obscured by contributions from other substances present in the biosystem.

In recent years, the integration of machine learning into Raman spectroscopic research has gained significant attention.^9−12^ Numerous machine learning algorithms have been applied to capture subtle spectral features and classify spectra.^10,11,13−20^ Among these, convolutional neural networks (CNNs) have proven particularly effective in processing raw spectra without the need for preprocessing or baseline correction.^10,11,18−20^ This is especially important for biological samples, where Raman spectra often exhibit strong baselines due to fluorescence and a weak signal-to-noise ratio, making preprocessing prone to artifacts.^10,18^ CNNs are well-suited for spectral analysis as they can detect local patterns, such as absorbance peaks or shifts, using convolutional filters. By adjusting filter or kernel sizes, CNNs effectively extract key spectral features. The end-to-end nature of CNNs, which integrates preprocessing, feature extraction, and classification, eliminates the need for manual feature engineering, thereby supporting more objective and reliable classification.^10,11,19,21−30^

However, existing studies that integrate CNNs with biomolecular spectra still face several challenges. First, common CNN architectures typically use a single kernel size for feature extraction, which can be limiting when dealing with spectral features of varying scales. Second, CNNs are often viewed as “black boxes”, making it difficult to interpret their classification decisions and understand how the model distinguishes between different spectra or biomolecule classes. Moreover, CNNs require large data sets for effective training, which is often difficult to obtain during the early stages of bioassay development.

To capture spectral features of varying sizes, we implemented a CNN with multiple parallel convolutional layers, using kernel sizes corresponding to characteristic Raman peaks. While the parallel structure has been used in previous studies,^31−33^ those works often lack a clear rationale for kernel size selection, which can lead to the omission of critical spectral features. Raman spectra from biomolecules typically exhibit peak features of different scales due to contributions from small molecules, molecular clusters, secondary structures, and other factors.^34−36^ These peaks reflect the physical dimensions associated with the vibrational modes of specific chemical bonds. We hypothesize that by accurately targeting these inherent features and minimizing the influence of noise and background signals, we can significantly enhance model accuracy.

Recent studies combining CNNs with biomolecular Raman spectrum classification have introduced feature map visualization techniques to assess whether important spectral features are being captured by the model,^25,26,37^ offering insights into which regions of the input spectra most influence the model’s predictions.^38−43^ These techniques have successfully identified characteristic peaks in simple systems with strong signals that are easily recognizable to the human eye. In this study, we aim to extend these visualization methods to more complex cell membrane systems, where the characteristic peaks of target molecules are embedded within intricate spectra, to reveal the critical decision-making criteria used by the model.

In this study, we developed a comprehensive approach to build a multiscale 1D-CNN for classifying the Raman spectra of cholera toxin B subunit (CTB) binding to the cell membrane platforms. To address the challenge of limited spectral data, we applied data augmentation using convex combinations. The multiscale CNN architecture was designed to extract features at different scales, enabling it to handle strong background signals and the variety of peaks typical in biomolecular spectra. By carefully selecting convolutional kernel sizes based on the scales of characteristic Raman peaks, we significantly improved the model’s ability to classify nonpreprocessed spectra. The model’s performance was validated through comparisons with other machine learning methods from the literature. Additionally, to gain insight into the model’s decision-making process, we utilized saliency heatmaps to visualize critical spectral regions influencing the CNN’s predictions, confirming its ability to identify key spectral features of CTB.

## Materials and Methods

### Materials

Calcium chloride (CaCl_2_), Dithiothreitol (DTT), HEPES (*N*-2-hydroxyethylpiperazine-*N*-2-ethanesulfonic acid), sodium chloride (NaCl), and paraformaldehyde (PFA) were purchased from Sigma-Aldrich (St. Louis, MO). Cholera toxin B subunit (CTB) and FAST-DiO (3,3′-dilinoleyloxacarbocyanine perchlorate) was purchased from Thermo Fisher Scientific (Waltham, MA). Poly(dimethylsiloxane) (PDMS) was purchased from Dow (Midland, MI). All chemicals were of analytical grade and used without further purification.

### Supported Cell Membrane Platform Preparation

Giant plasma membrane vesicles (GPMVs) derived from HeLa cells were utilized to create a supported cell membrane platform. Prior to GPMV induction, HeLa cells were washed 3 times with GPMV buffer (10 mM HEPES, 96.7 mM NaCl, and 2 mM CaCl_2_, pH 7.4) in culture dishes. Following this, a vesiculation reagent (25 mM PFA, 2 mM DTT in 1 mL of GPMV buffer) was added to the dish and incubated at 37 °C for 1 h to induce the formation of GPMVs. GPMVs were ruptured on a triangular gold-deposited chip to form planar cell membrane patches. The deposition detail can be found in our previous study.^44^ To identify the locations of the GPMV patches on the substrates, the cells were labeled with 1 mg/mL FAST-DiO for 10 min at 4 °C before vesiculation.

For the CTB treated samples, the cell membrane samples were treated with 1, 10, or 100 ng/mL of CTB in GPMV buffer and incubated for 10 min, followed by the GPMV buffer wash. For the non-CTB treated samples, the cell membrane samples were treated with GPMV buffer with no CTB for 10 min, followed by the GPMV buffer wash.

### Data Acquisition

The CTB treated and non-CTB treated samples were placed on the stage of a Raman microscope. Fluorescence microscopy was used to focus on the dyed membrane patches. Raman spectra were recorded using an inVia confocal Raman microscope (Renishaw, U.K.) equipped with a He–Ne laser for 633 nm excitation. The laser power was set to 10% of 14.1 mW. To improve the signal-to-noise ratio, all Raman spectra were accumulated 30 times. Data acquisition was performed using Renishaw WiRE 5.0 software, and a charge-coupled device (CCD) detector was utilized for capturing the spectra.

### Multiscale 1D-CNN Training and Evaluation

The multiscale one-dimensional (1D)-CNN was optimized using 5-fold cross-validation. The data set was initially divided into two halves, with one-half reserved for testing. From the remaining half, one-fifth of the spectra from each class were designated as the validation set, while the rest were used for training. Data augmentation was applied using convex combinations^45^ within identical classes. The distribution of training, validation, and test data under different conditions for each iteration is summarized in Table 1.

**Table 1:** Total Number of Spectra, Along with Counts for Original Training, Validation, and Test Data Sets before and after Augmentation

condition	training and validation	training and validation (augmented)	test	test (augmented)
CTB (1 ng/mL)	22	2079	21	1890
non-CTB	22	2079	21	1890

Table 2 lists the tuning ranges of the model hyperparameters. The model training was performed by Adam optimizer. Early stopping was applied to determine the end of training with a patience of ten. Binary cross-entropy was selected as the loss function, which calculated the loss between ground truth and predictions, and then the loss was returned for backpropagation.

**Table 2:** Tuning Ranges of Model Hyperparameters

hyperparameter	tuning range	hyperparameter	tuning range
kernel size	listed in Table3	dropout rate	[0, 0.1, 0.2, 0.3, 0.4, 0.5]
number of parallel convolutional layer	[1, 2, 3, 4, 5]	node	[16, 32,···,2048, 4096]
channel	[16, 32, 64, 128]	learning rate	10^–5^ ∼ 10^–2^

All the runs were conducted using Python v3.10.0 and TensorFlow v2.10.1 on a PC system with NVIDIA GeForce RTX 4090 with 128GB DDR5 and CPU of Intel core i9–14900 K.

### Saliency Scores and Heatmaps of Gradient on Activation Map (Grad-AM)

Class saliency scores (*s*_*j*_^*c*^) were calculated by evaluating the gradients of the final output for a given class (*y^c^*) with respect to the activation map at each wavenumber (*A*_*j*_^*k*^). In this notation, the superscript *c* denotes the class index, the subscript *j* indicates the wavenumber index, and the superscript *k* represents the index of the activation map.

The derivative of the final class output (*y^c^*) with respect to the value at a specific wavenumber within a particular activation map (*A*_*j*_^*k*^) was computed. This derivative was then multiplied by the sign of the activation map value at that wavenumber to correct for the impact of inverted reflections in the activation maps, which is shown in Figure S4. In such cases, an increase in the activation value correspond to a decrease in the mirrored feature, causing the true positive influence of a feature at a specific wavenumber to be misinterpreted as a negative influence.

The adjusted derivatives across all activation maps for a specific wavenumber were summed to quantify the contribution of the feature at the wavenumber to the classification result. The saliency scores were normalized by the sum (*S^c^*) across all wavenumbers. The saliency score for wavenumber index *j* in class *c* is defined as
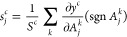
where 

The Grad-AM heatmap (*L*_grad–AM_^c^) is then computed as
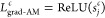
where a rectified linear unit (ReLU) operator is applied to set negative values to zero and leave positive values unchanged, ensuring non-negative pixel values in the heatmap visualization.

### Saliency Scores and Heatmaps of Gradient on Input (Grad-Input)

Class saliency scores of gradients on input (*w*_*i*_^*c*^) were calculated from the derivative of the final output of a specific class (*y^c^*) with respect to the input spectrum (*X_i_*).^46^ These scores were normalized by the sum (*W*^*c*^) across all wavenumbers. The class saliency score for wavenumber *i* in class *c* is defined as
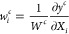
where 

The Grad-input heatmap (*L*_grad–input_^*c*^) is computed as
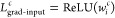


### Heatmaps of Grad-CAM

Grad-CAM heatmaps were generated by linearly combining weight contributions and activation maps.^47^ First, the test spectrum was fed into the trained model, and *k* activation maps were extracted from the convolutional layers. The partial derivatives of each wavenumber in the *k*th activation map (*A^k^*) was calculated with respect to the class output scores (*y*^*c*^). These partial derivatives at different wavenumbers in the specific activation map were then summed and normalized, yielding the weight contribution of the *k*th activation map to the classification result. The weight contribution (α_*k*_^*c*^) of the *k*th activation map for the class *c* is defined as
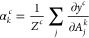
where 

The Grad-CAM heatmap (*L*_grad–CAM_^*c*^) is computed as
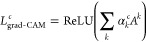


## Results and Discussion

### Use of Cell Membrane Platform to Detect CTB via Raman Spectra

**Figure 1:**
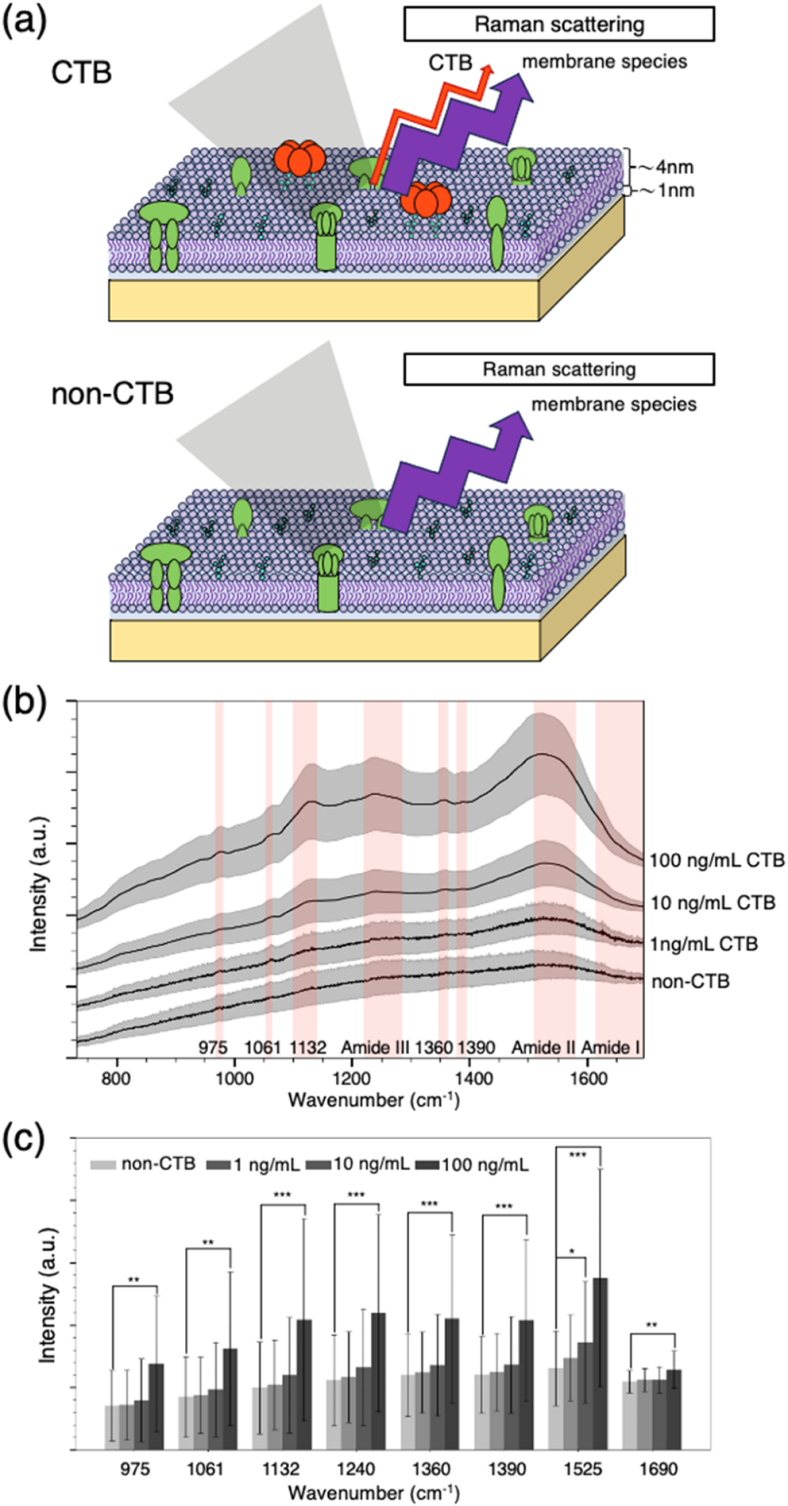
(a) Schematic illustration of the CTB detection platform using a cell membrane sensor. The top section shows the CTB-treated scenario, where SERS signals arise from both CTB molecules and various components within the cell membrane patch. The lower section illustrates the nontreated scenario, where SERS signals originate solely from membrane components in the cell membrane patch. (b) The SERS spectra for samples treated with various CTB bulk concentrations (100, 10, and 1 ng/mL) and nontreated samples, with shaded areas representing one-third of the actual standard deviations. Red bands highlight peaks or bands significantly growing with treated CTB concentrations. (c) Statistical analysis of spectral differences between CTB-treated and non-CTB samples. Bar graphs show the mean intensities and standard deviations at selected peaks or bands that appeared to increase with CTB concentration. Symbols indicating statistical significance: * (*p* ≤ 0.05), ** (*p* ≤ 0.01), *** (*p* ≤ 0.001).

We employed cell membrane patches as sensing elements to assay the cholera toxin B subunit (CTB), demonstrating the potential of a cell membrane-based sensor for toxin detection, as illustrated in Figure 1a. Giant plasma membrane vesicles (GPMVs) were extracted from HeLa cells to form supported cell membrane patches on a surface-enhanced Raman spectroscopy (SERS) chip. GPMVs, derived directly from cells, preserve the compositional complexity and membrane protein content characteristic of biological membranes.^48−50^ The lipids and proteins in the cell membrane could serve as bioelements, capturing pathogens, toxins, or other substances to the membrane. The chemical identity of the bound species was detected via Raman spectroscopy. CTB was chosen as a model toxin in this study due to its known interaction with monosialotetrahexosylganglioside (GM1) in native cell membranes.^51^

Figure 1a illustrates our cell membrane detection system and explains why CTB signals are weak and challenging to detect. The cell membrane consists of a single lipid bilayer with associated biomolecules, and CTB binds to GM1 receptors within the membrane. The GM1 content is estimated to be approximately 0.05 mol %,^52,53^ resulting in surface densities of CTB on membrane patches of 50, 208, and 390 molecules within the laser detection area (0.785 μm^2^) for CTB concentrations of 1 ng/mL, 10, and 100 ng/mL, respectively (detailed estimation in SI). This CTB density is comparable to or even lower than that of lipids and other biomolecules in the membrane, leading to a relatively weak signal. Additionally, variations in lipid and protein composition across different membrane patches introduce significant fluctuations in the signals of cell membrane patches, further complicating CTB detection. As a result, the weak CTB signal can be obscured by the membrane’s heterogeneous background, making it difficult to identify CTB through simple subtraction or comparison methods.

Figure 1b presents the normalized raw SERS spectra for samples with and without CTB treatment. The dark lines represent the average spectra across 43 measurements for each condition, while the gray shading indicates the standard deviation, highlighting significant variability among samples. Our objective is to distinguish and classify the 1 ng/mL CTB-treated sample from the non-CTB sample, as 1 ng/mL is considered a physiologically relevant CTB concentration.^54^ However, visual inspection alone does not reveal clear peak differences between these two conditions. To identify spectral features associated with CTB, we also examined samples treated with higher CTB concentrations (10 and 100 ng/mL) to observe which peaks or bands increase with increasing CTB levels.

We observed that peaks with an approximate bandwidth of 20 cm^–1^, centered around 975, 1061, 1132, 1360, and 1390 cm^–1^, increase with rising CTB concentrations and may correspond to amino acid spectral signatures. In aqueous solution, amino acids such as Ile, Leu, and Ser exhibit peaks near 975 cm^–1^,^55^ while Lys, Met, and Val show peaks around 1061 cm^–1^,^55^ which also correspond to C–N and C–C stretching modes.^56^ Additionally, amino acids including Ala, Lys, Arg, Asp, Glu, His, Met, Trp, and Val display peaks near 1360 cm^–1^,^55^ whereas Ala and Glu exhibit peaks around 1390 cm^–1^.^55^ These peaks are more pronounced in CTB-treated samples, aligning with the high abundance of Ala, Ile, and Lys in CTB, as detailed in Table S1 (SI). Furthermore, bands at approximately 1265, 1550, and 1650 cm^–1^, corresponding to Amide III, Amide II, and Amide I protein structures, respectively, also show differences between CTB-treated and untreated samples. The presence of CTB, as an additional protein, likely contributes to the enhanced signals in these regions.

We further investigated whether the peaks or bands that appeared to increase with CTB concentration, as identified by visual inspection, could be statistically distinguished between CTB-treated and non-CTB samples. As shown in the bar graphs in Figure 1c, the spectra of 1 ng/mL CTB-treated and non-CTB samples did not exhibit statistically significant differences at any of these spectral locations. Only at 100 ng/mL CTB treatment, the intensities at these peaks became statistically distinct from the non-CTB samples. This finding underscores the challenge of identifying low-surface-density CTB in individual spectra. To address this challenge, we developed a convolutional neural network (CNN) capable of objectively distinguishing CTB signals, even when they are not statistically different from non-CTB samples due to the small number of target molecules and the highly variable biological background.

### Multiscale 1D-CNN for Raman Spectrum Classification

Biological samples are inherently complex, containing a diverse array of biomolecules, and organic compounds. This complexity often results in Raman spectra with peaks of varying widths, a feature also observed in our cell membrane system (Figure 1b). Broad peaks typically arise from biological molecules such as proteins, lipids, and nucleic acids existing in diverse environments, which leads to inhomogeneous broadening. Biomolecules may experience distinct interactions, such as hydrogen bonding, solvent effects, or structural disorder, resulting in a spread of vibrational frequencies. Additionally, the flexible and dynamic nature of biological structures can contribute to vibrational coupling, further broadening peaks. In contrast, narrow peaks are usually associated with more uniform, well-defined molecular structures or bonds that undergo less variation in their local environments, leading to more discrete vibrational modes. We hypothesized that capturing all of the important peak features with different sizes could significantly improve detection accuracy. To achieve this, we developed a CNN with a parallel architecture of convolutional layers using different kernel sizes to extract peak features of varying sizes.

**Figure 2:**
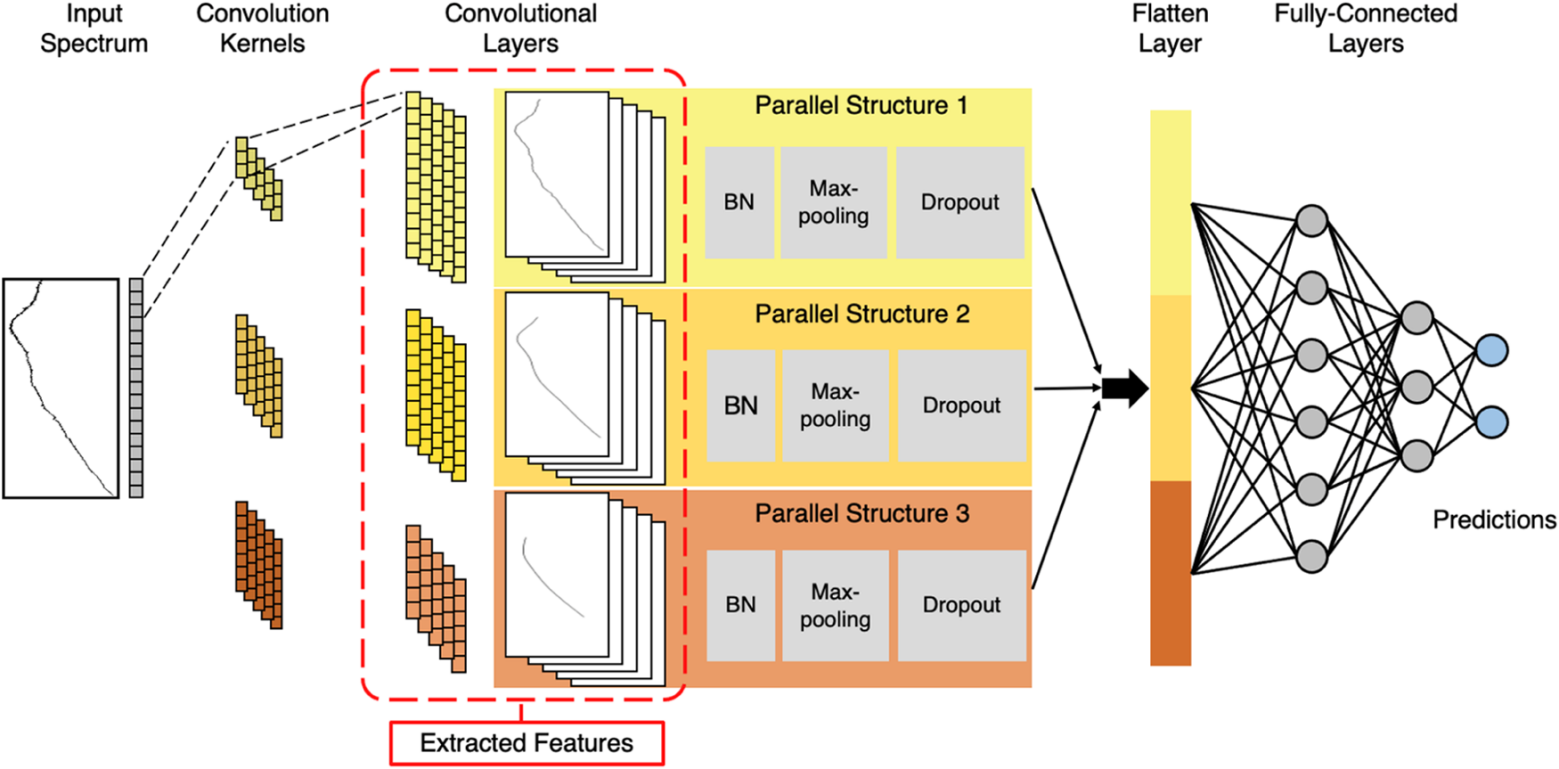
Schematic of the multiscale 1D-CNN architecture for spectral classification. The input spectra are processed through parallel 1D convolutional structures, each designed to extract features with different size scales. These parallel structures consist of batch normalization (BN), max-pooling, and dropout layers, which help in feature stabilization, dimensionality reduction, and overfitting prevention, respectively. The extracted features from each parallel structure are then flattened and fed into fully connected layers to generate the final predictions. The activation maps with extracted features after convolution are displayed in the red dashed box.

Figure 2 illustrates the architecture of the proposed multiscale 1D-CNN. The model consists of several parallel structures, each containing convolution, batch normalization, pooling, and dropout layers. These parallel structures are designed to extract features at different scales from the input spectra. The outputs from these parallel paths are concatenated in a flatten layer and then passed through fully connected layers to generate the final prediction. This use of parallel structures during feature extraction enables the model to capture a wide range of spectral features. The number of kernels, the quantity of each kernel size, and the specific kernel sizes used in the convolutional layers were fine-tuned through 5-fold cross-validation, with details provided in the next subsection and SI (Table S2). To mitigate overfitting, we incorporated pooling, batch normalization, and dropout layers,^57,58^ which were also tuned as hyperparameters. The architecture concludes with fully connected layers for classification, leveraging features extracted by earlier layers. The dropout rates and number of nodes in the connected layers were also optimized via 5-fold cross-validation.

### Convolutional Kernel Size Selection for Multiscale Feature Extraction

The size scale of the extracted feature in CNNs is determined by the size of the convolutional kernel.^28^ Although we have some knowledge of the characteristic peak sizes for the vibration of bonds associated with amino acids and secondary structures, the optimal choice of convolutional kernel sizes remains uncertain. Therefore, selecting the number of parallel structures and the specific kernel sizes to use poses a significant challenge. We planned to treat these as hyperparameters that require tuning. However, the vast number of possible combinations makes exhaustive hyperparameter optimization impractical. To address this, we grouped kernel sizes ranging from 3 to 367 pixel intervals based on their influence on peak feature extraction. We input the actual spectral data points—intensity at each pixel of our spectrometer detector—into the model for analysis. In this study, a wavenumber range of 1170 cm^–1^ was recorded using 1009 pixels, resulting in a pixel width equivalent to 1.107 cm^–1^ per pixel. To enhance the broader applicability and physical relevance of our results, we also converted kernel sizes from pixel-based values to wavenumber-based intervals, as shown in Figure S2.

To better understand the effect of different kernel sizes, we used a moving average process as a reference, given its equivalence to a convolution with a unit-value kernel with zero standard deviation. By applying this type of convolution with different sizes of unit-value kernels to a spectrum, we could observe how kernels of varying sizes smooth out features at different scales (more details provided in the SI, Figure S3). As expected, increasing kernel sizes progressively smoothed out finer spectral details. Smaller kernels preserved narrower features, while larger kernels captured broader trends.

**Figure 3:**
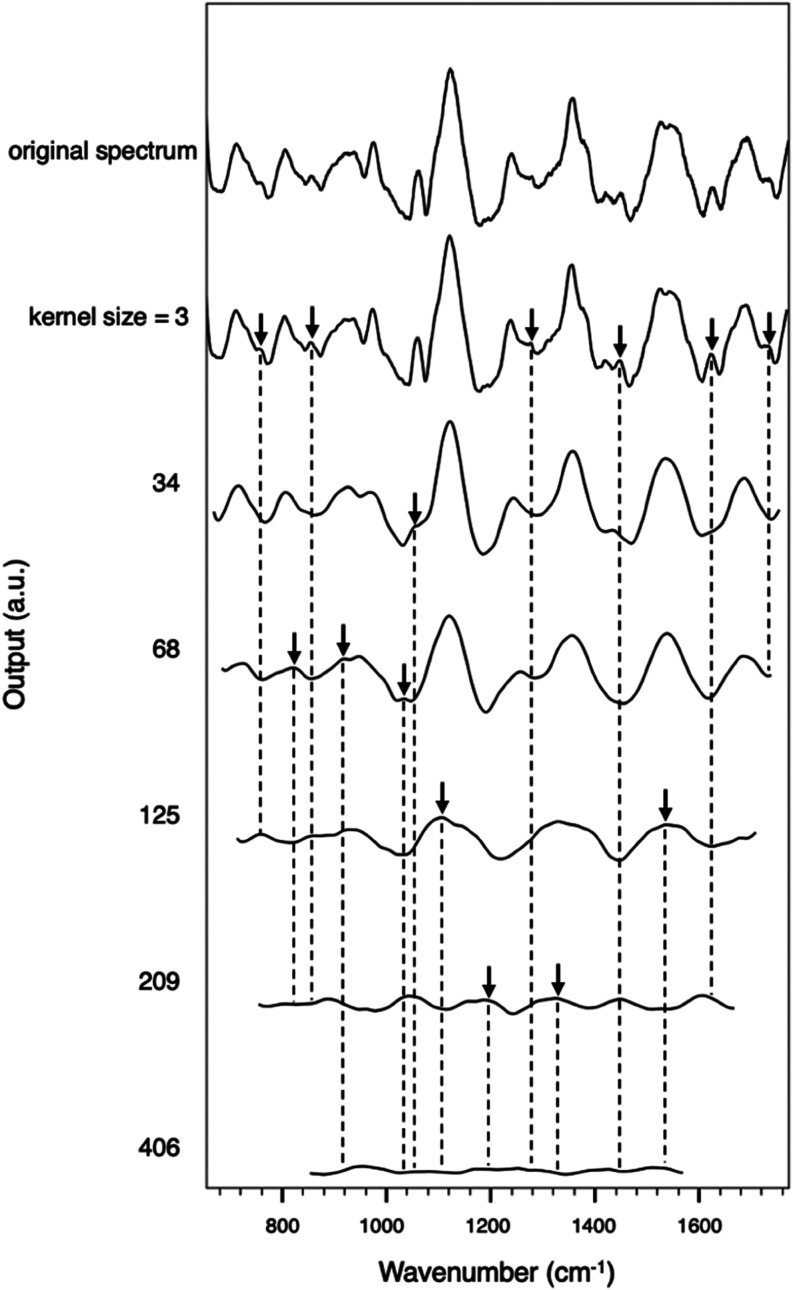
Postprocessed spectra from the SERS spectrum of a 100 ng/mL CTB-treated sample, generated by convolutional layers using unit-value kernels at the cutoff sizes. The figure highlights the largest kernel size at which specific peaks remain visible. Arrows indicate peaks that persist at each cutoff size, illustrating how increasing kernel sizes progressively eliminate finer spectral details. These cutoff sizes define five kernel levels (A–E, detailed in Table 3), which guide kernel selection for feature extraction. The cutoff kernel sizes are 3 (3 cm^–1^), 31 (34 cm^–1^), 61 (69 cm^–1^), 113 (125 cm^–1^), 189 (209 cm^–1^), and 367 (406 cm^–1^).

Figure 3 illustrates the postprocessed spectra at the size cutoffs of unit-value kernels, showing the largest kernel size at which specific types of peaks remain visible. These cutoffs could act as thresholds that adjust the scale of feature extraction. Using these cutoff sizes, we defined five levels of kernel sizes, as shown in Table 3, labeled from A to E, for use in the kernel selection. Beyond each cutoff size, the corresponding peaks disappear at the subsequent level, while the remaining peaks stay visible. The arrows in Figure 3 indicate peaks that remain visible at the cutoff size, demonstrating how kernels beyond each size lead to the disappearance of features with particular characteristic sizes. Kernel sizes within the same level (between two cutoffs) could exhibit similar smoothing effects.

**Table 3:** Ranges of Kernel Sizes for Hyperparameter Tuning

level number	range of kernel size (pixel interval)	range of kernel size (cm^–1^)
level A	[3, 5, ···, 27, 29]	[3, 5, ···, 30, 32]
level B	[31, 33, ···, 57, 59]	[34, 37, ···, 64, 66]
level C	[61, 63, ···, 109, 111]	[68, 70, ···, 120, 123]
level D	[113, 115, ···, 185, 187]	[125, 127, ···, 205, 207]
level E	[189, 191, ···, 365, 367]	[209, 211, ···, 404, 406]

### Analysis of Data Augmentation

Convolutional neural networks (CNNs) are data-hungry models, and obtaining a sufficient number of original spectra for training can be challenging for biological samples. To address this limitation, data augmentation becomes a crucial strategy.^27^ We normalized the spectra to a range of 0—1 and applied a convex combination technique,^45^ which preserves the essential spectral features from the original data. The augmentation is calculated as follows:
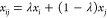
where *x*_*i*_ and *x*_*j*_ are different spectra represented as vectors, and λ is the convex combination factor, ranging from 0 to 1.

We hypothesized that appropriate data augmentation would improve model accuracy by providing more training instances, allowing better weight adjustments. However, augmentation could be limited by the overall information content of the original data set—excessive augmentation might not add meaningful variability and could instead increase computational load or lead to overfitting. Thus, we treated λ as a hyperparameter to be tuned. Table 4 shows the search space for λ values. With the current number of spectra at 22, applying augmentation with λ = 0.5 increased the data set to 231 spectra after removing duplicates. The augmentation possibilities expand further with the inclusion of additional λ values, as shown in the lower rows of Table 4.

**Table 4:** Classification Performance of CTB-Treated and Nontreated Samples for Different Augmentation Factor Combinations^a^

augmentation factor combination	# of training spectra	accuracy (%)	sensitivity (%)	specificity (%)	precision (%)
no augmentation	22	47.67 ± 4.05	74.80 ± 35.50	20.54 ± 27.87	44.26 ± 11.07
[0.5]	231	96.44 ± 1.82	98.06 ± 0.37	94.83 ± 3.75	95.12 ± 3.44
[0.1, 0.2,···, 0.9]	2079	97.40 ± 0.82	98.53 ± 0.80	96.26 ± 1.83	96.38 ± 1.74
[0.01, 0.02,···, 0.99]	22,869	96.94 ± 0.73	98.01 ± 0.19	95.86 ± 1.41	95.97 ± 1.34

a The Results are Obtained by Using a 1-D CNN with Single Kernel Size of 81 (90 cm^–1^).

As illustrated in the right panel of Table 4, the CNN’s performance improved significantly with data augmentation. The best results were achieved using a combination of augmentation factors [0.1, 0.2,···, 0.9], which yielded an accuracy of 97.40%, sensitivity of 98.53%, specificity of 96.26%, and precision of 96.38%. In contrast, the model without augmentation had much lower accuracy (47.67%) and poorer performance across all other metrics, emphasizing the importance of data augmentation in training robust models in this limited data case. Even simpler augmentation strategies, such as using a single factor (λ = 0.5), produced noticeable performance gains, indicating that even basic augmentation can positively contribute to the model’s generalization ability. These results suggest that carefully selecting augmentation factors can significantly enhance classification performance, particularly in situations where data is limited.

### Comparison of Multiscale 1D-CNN with Other Machine Learning Algorithms

We performed hyperparameter tuning on our proposed multiscale 1D-CNN (Figure 2), evaluating configurations with one to five parallel convolutional structures, using kernel sizes across the five levels previously discussed. Recognizing that not all types of spectral peaks may be crucial for classification, we systematically tested structures with varying numbers of parallel convolutional layers—ranging from one to five—to identify the configuration yielding the best performance. In the five-parallel configuration, one kernel size was selected from each of the five levels. In the four-parallel configuration, one kernel size was selected from each of any four levels, and this pattern continued for the remaining configurations.

**Figure 4:**
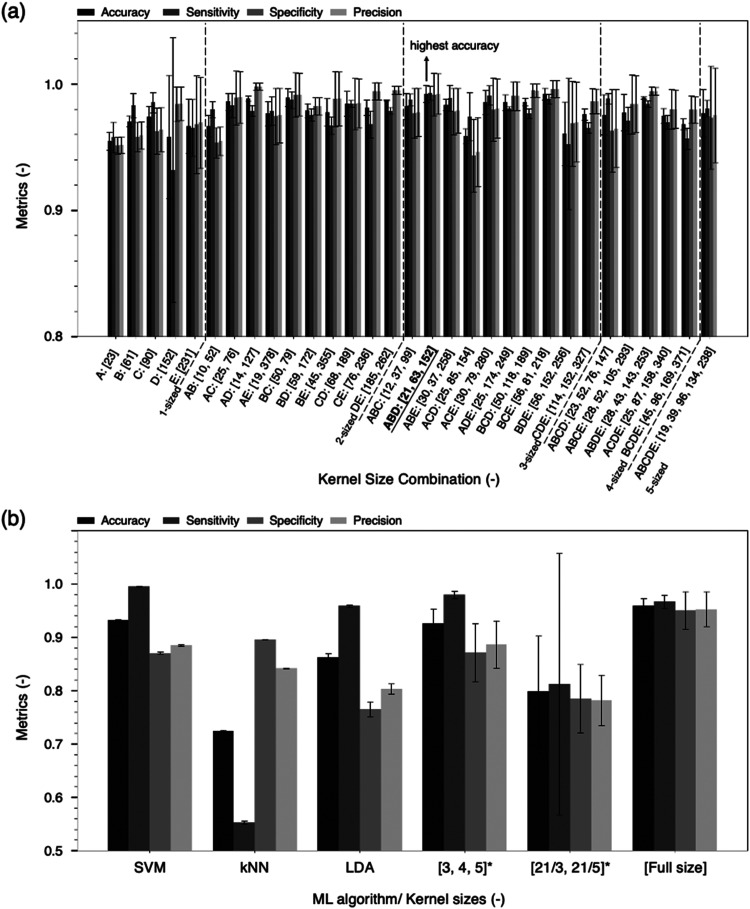
Classification performance of 1 ng/mL CTB-treated and nontreated samples for (a) our proposed CNN structures with various kernel size combinations. Here, we list only the performance values achieved by the best-performing kernel size(s) for each possible level combination. The tuned hyperparameters are listed in Table S2. (b) The performance for different machine learning algorithms and CNN structures proposed by other studies. The kernel sizes here are expressed in cm^–1^, converted from pixel intervals (odd integers) and rounded to the nearest integer. The kernel sizes are in pixel intervals as originally reported in the respective studies and marked with *. In the kernel size combination [21/3, 21/5], the multiscale CNN includes two parallel structures. One structure uses a kernel size of 21 followed by a kernel size of 3, while the other uses a kernel size of 21 followed by a kernel size of 5.

Figure 4a illustrates the optimal kernel size combinations for different numbers of parallel structures. Notably, the three-parallel structure, utilizing kernel sizes of 21, 63, and 152 cm^–1^ at levels A, B, and D, achieved the highest performance, with a test accuracy of 99.22%, sensitivity of 99.27%, specificity of 99.16%, and precision of 99.20%. We find the selection of kernels at levels A, B, and D to be reasonable, as they align with observable biomolecular Raman peak occurrences in aqueous solutions. The Raman peak widths of biomolecules in solution vary due to factors such as molecular interactions with water, hydrogen bonding, and dynamic motions.^55,59−61^ Typically, the full-width at half-maximum (fwhm) for amino acid Raman peaks ranges from 10 to 30 cm^–1^.^55,59^ Peaks within the amide band or regions where multiple amino acids have adjacent peaks range from 40 to 70 cm^–1^,^55^ while broader aggregated peaks of 120–150 cm^–1^ can also be observed when amino acids dissolve in solution.^55,59^ These peak ranges closely correspond to levels A, B, and D in the defined kernel size ranges, supporting the validity of the tuned kernel combination of 21, 63, and 152 cm^–1^.

To validate the effectiveness of this parallel convolutional design in classifying biomolecular spectra, we compared our proposed model against other machine learning algorithms. As shown in Figure 4b, traditional methods such as SVM, LDA, and kNN demonstrated inferior performance. This underscores the superior capability of our multiscale 1D-CNN to effectively handle the complexities of biological spectra.

We also compared our multiscale 1D-CNN to other multiscale models from previous studies. Models proposed by Ding et al.^32^ and Tang et al.^62^ lacked clear criteria for selecting kernel sizes. When applied to our data set using their kernel combinations [3, 4, 5] and [21/3, 21/5], these models underperformed relative to our parallel convolutional model, which uses kernel sizes tailored to relevant peak widths. Additionally, Deng et al.^31^ proposed a CNN that employed all range of kernel sizes, which is supposed to capture full-scale features. However, on our data set, this model also exhibited lower classification performance. This could be attributed to their use of channel reduction to lower computational demands, potentially losing valuable spectral features extracted by earlier layers. Furthermore, our results in Figure 4a indicate that increasing the number of parallel structures does not necessarily enhance accuracy. This may be due to the inclusion of excessive, less significant information, which can dilute the contribution of critical features during training and reduce the overall effectiveness of the model.

### Visualization of Spectrum Classification with Saliency Heatmaps

**Figure 5:**
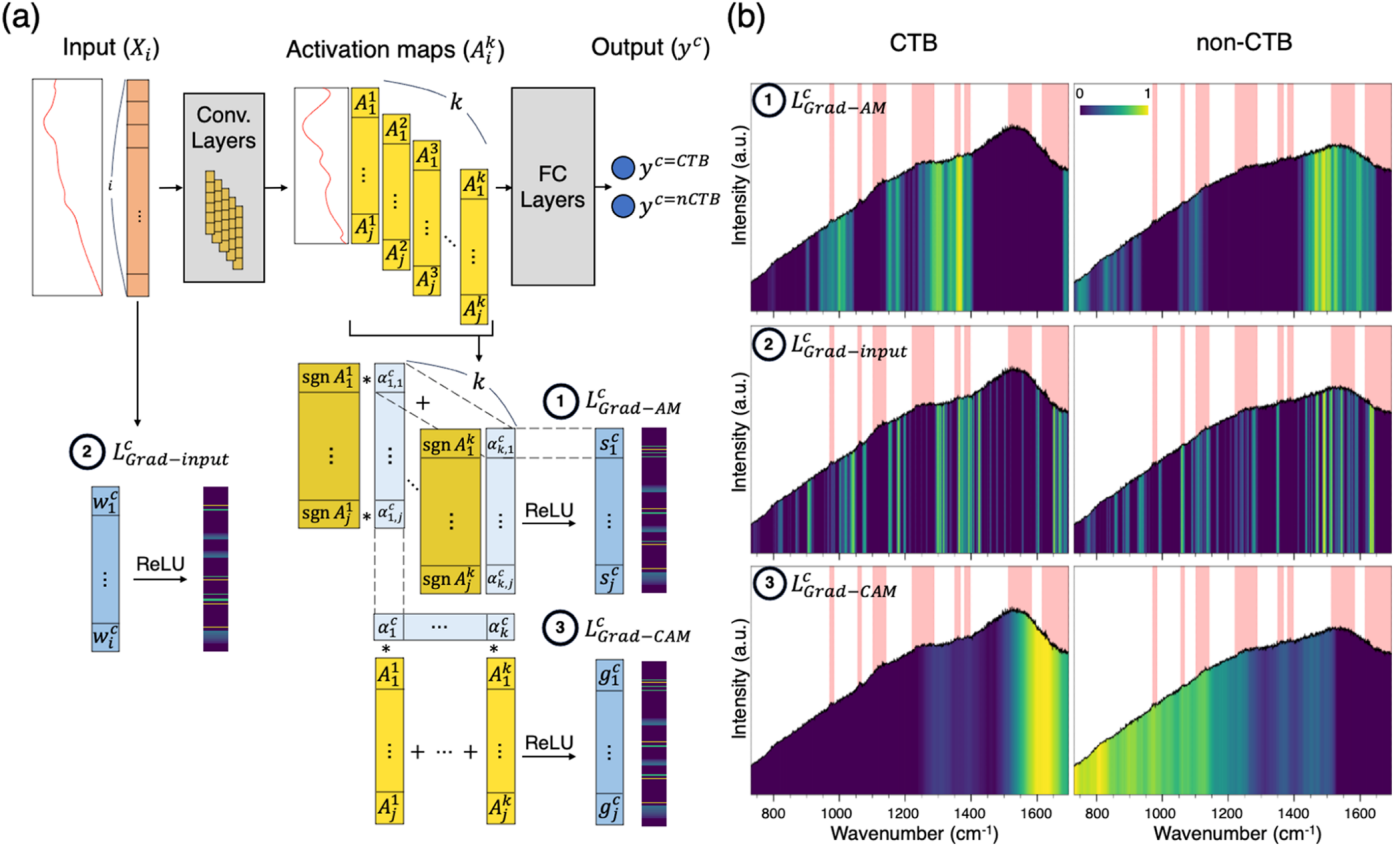
(a) Schematic pipeline for calculating (1) *L*_grad–AM_^*c*^, (2) *L*_grad–input_^*c*^, and (3) *L*_grad–CAM_^*c*^. Herein, . The remaining details are in Materials and Methods. (b) The corresponding saliency heatmaps of 1 ng/mL CTB-treated and nontreated spectra, illustrating the importance of each wavenumber in identifying key spectral features. The black lines are the averaged SERS spectra of 1 ng/mL CTB-treated and nontreated samples, respectively. The red bands label noticeably stronger peaks observed in the CTB-treated samples compared to the nontreated samples. The heatmaps are calculated from the mean of all original test spectra before data augmentation.

To gain deeper insights into the decision-making process of our CNN, we used saliency heatmaps to visualize the critical spectral regions influencing the model’s predictions. We first employed two common visualization methods—Grad-Input and Grad-CAM—to examine the CNN’s classification behavior. As shown in Figure 5a, Grad-Input heatmaps were generated by calculating the gradient of the output for a specific class (*y*^*c*^) with respect to the signal intensity at each wavenumber in the input spectrum.^46^ This method highlights how changes in intensity at each wavenumber in the input spectrum impact the model’s predictions. Grad-CAM, another popular visualization technique, identifies the contribution of each activation map to the model’s predictions. While Grad-CAM has proven effective in image classification tasks,^47^ its application to spectral data is less explored. In Grad-CAM, heatmaps are generated by linearly combining activation maps with their corresponding weights,^47^ where each weight is obtained by summing the partial derivatives of the class score (*y*^*c*^) with respect to the activation map. However, traditional Grad-CAM can pose a potential issue by applying a weighted sum across the entire activation map, which may inadvertently amplify some spectrum regions with nonessential peaks. To address this issue, we developed a new method for assessing the importance of peak features in classification. Similar to Grad-CAM, our approach evaluates how variations in intensity within the activation maps (rather than directly from the raw input spectra) influence the classification outcome. Unlike Grad-CAM, our method calculates the score for each wavenumber by summing the derivatives at that wavenumber across different activation maps, minimizing the risk of overemphasizing low-impact spectrum regions.

In our method, gradients were evaluated based on the activation maps rather than the raw input spectra, given that peaks in Raman spectra have finite widths, and intensity changes at a single wavenumber may not directly indicate a significant peak. Activation maps are designed to capture and emphasize peak features, incorporating neighboring information through convolutional operations. The extent of this neighboring information is determined by kernel sizes, which are optimized during training. Therefore, we believe that examining how variations in intensity within the activation maps influence the spectrum classification outcome may provide a more reliable measure of feature importance.

Figure 5b displays the heatmaps generated by three different saliency methods. Brighter colors indicate higher saliency scores at specific wavenumbers, highlighting spectral features emphasized during classification. The top panel shows the saliency heatmaps from Gradient on Activation Map (*L*_grad–AM_^*c*^) for both 1 ng/mL CTB-treated and nontreated classes. For the CTB-treated class, *L*_grad–AM_^*c*^ emphasizes broad regions around 945–1045, 1145–1200, 1245–1400, and 1680–1700 cm^–1^. These highlights align with several spectral features marked in red in our CTB-treated raw data, including peaks at 975, 1360, 1390 cm^–1^, amide III around 1300 cm^–1^, and amide I around 1685 cm^–1^.

We note that some highlighted wavenumbers, such as 903, 1152, and 1177 cm^–1^, lack observable peaks in the averaged raw data of 1 ng/mL CTB-treated samples. This may suggest that the CNN can identify spectral features less apparent to the naked eye. In addition, certain peaks observed in the 1 ng/mL CTB-treated raw data, such as those at 1132, 1061 cm^–1^, and in the amide II region around 1550 cm^–1^, were not strongly highlighted. We found that these regions are highlighted in the nontreated class instead. We think this is because both classes have peaks in these spectrum regions (as peaks at 1132 and 1061 cm^–1^ are associated with general amino acid types), and the intensity variation among the CTB-treated samples is larger than that of the non-CTB samples. Therefore, the existence of these peaks was not emphasized during the CTB-treated class classification. We will explain these inconsistencies between the highlighted regions and the observable features in the spectra in the later subsections.

The middle panel shows the heatmaps from Grad-Input Map (*L*_grad–input_^c^) for both 1 ng/mL CTB-treated and nontreated classes. *L*_grad–input_^c^ highlights gradients of the input spectrum with respect to the model’s output. The highlighted lines correspond to several spectral features in our CTB-treated raw data, including peaks at 975, 1360, 1390 cm^–1^, amide III around 1300 cm^–1^, and amide I around 1685 cm^–1^. The highlighted regions follow similar trends to those in *L*_grad–AM_^c^ for both classes. The major difference is that *L*_grad–input_^c^ only highlight the importance at each pixel, making it difficult to discern the significance of spectral peaks or bands with specific widths. For instance, at 1000 and 1300 cm^–1^, while *L*_grad–input_^c^ highlights some thin lines near these regions, *L*_grad–AM_^c^ provides broader, more intense coverage, potentially relevant to the spectral peaks or bands.

The bottom panel presents Grad-CAM heatmaps (*L*_grad–CAM_^c^) for both 1 ng/mL CTB-treated and nontreated classes. The *L*_grad–CAM_^c^ for the CTB-treated class shows a strong broad highlight between 1500 and 1650 cm^–1^, and weak highlight in the region of 1300, 1360, and 1390. The weakly emphasized region is consistent to those shown in *L*_grad–AM_^c^ and *L*_grad–input_^c^. However, the strong emphasis between 1550 and 1700 cm^–1^ were not found in the other two.

To investigate the reason, we examined all activation maps with their derivatives with respect to the classification outcome, as shown in Figure S4. The score for an activation map is determined by summing the derivatives across all wavenumbers. For instance, in kernels #5, #11, #17, #25 and etc. in Figure S4(a), positive derivatives mainly appear below 1450 cm^–1^, with a negative influence between 1500 and 1650 cm^–1^. However, the overall score for the activation map is positive, so the entire map is multiplied by a positive score, causing the negatively influencing regions to register as positive influences. As a result, some highlighted regions, such as the region between 1500 and 1650 cm^–1^ may not actually contribute positively to classification. This reduced interpretability about the spectra stem from Grad-CAM attributing significance to all areas within highly scored activation maps, which can lead to the erroneous identification of less relevant regions as crucial.

### Correlation between Grad-AM Saliency Score and Sample Variation

**Figure 6:**
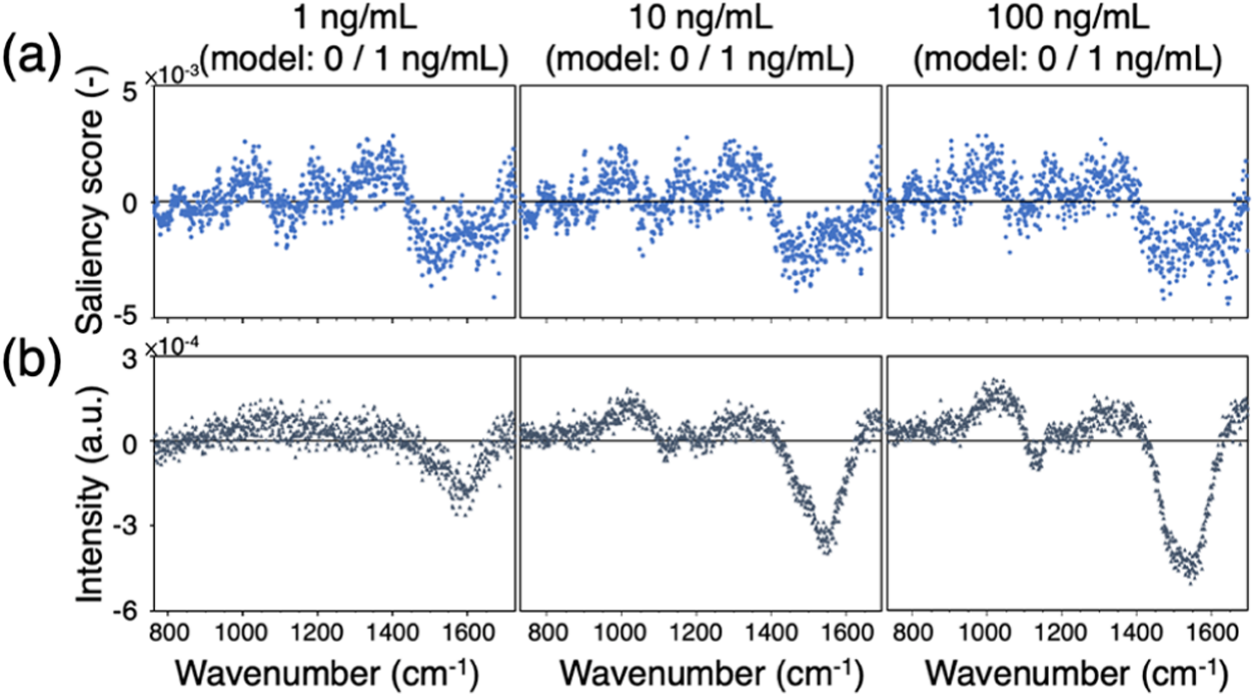
Comparison of Grad-AM saliency scores and sample variations. (a) Grad-AM saliency scores when test data from 1, 10, and 100 ng/mL CTB-treated samples were input into a model trained with the 1 ng/mL CTB-treated and non-CTB classes. (b) Difference in normalized standard deviation between CTB and non-CTB samples at each wavenumber, calculated by subtracting the normalized standard deviation of CTB samples from that of non-CTB samples.

From Figure 5, it is evident that Grad-AM highlights certain characteristic peaks associated with CTB. However, it is unclear why only specific peaks are highlighted while others are not. Since the model is trained for classification, regions with significant sample variation and lack of consistency are unlikely to be used as reliable decision-making features. To investigate this, we calculated the normalized standard deviation of the intensity at each wavenumber to represent the relative variation at each position. We then subtracted the normalized standard deviation of CTB samples from that of non-CTB samples. The resulting differences were plotted against the wavenumber, as shown in Figure 6b. A positive difference indicates that the signal variation at a given wavenumber is larger in non-CTB samples compared to CTB samples, suggesting that CTB samples exhibit more consistent signals in those regions. Conversely, a negative difference implies that CTB samples have greater variation at those wavenumbers, making it harder to classify CTB samples based on signals from these areas.

When comparing the difference plot to the Grad-AM saliency score plot (Figure 6a), we observed a strong correspondence: regions with positive differences generally align with positive contributions in the saliency score, while regions with negative differences correspond to negative contributions. Given that low-concentration CTB spectra exhibit more noise, we also compared the difference and saliency score plots for two high-concentration CTB conditions. These comparisons revealed a similar trend, further supporting the idea that the Grad-AM saliency score reflects the impact of sample variation.

This observation helps explain why no highlighted regions are observed in the 1450–1650 cm^–1^ range. Although this region shows a significant intensity increase with CTB concentration, a closer examination reveals subtle shape differences between samples at various concentrations, with sample variation in this range being larger than in other regions. Since the peak corresponds to CH/CH_3_ deformation and C–C stretching,^63^ which often appear as broad bands, we hypothesize that the laser may cause damage to membranes and proteins, generating or changing short carbon chains whose signals are then detected. The extent of this damage may vary between experiments, resulting in increased sample variation in this region. As a result, despite the intensity increase, the signal growth in this region may not reliably indicate the presence of CTB.

While the positive and negative regions in the two plots mostly align, there are notable differences in the relative magnitudes of the saliency scores and variation differences. Additionally, near-zero regions exhibit occasional mismatches in polarity, such as those around 750, 820, 930, and 1105 cm^–1^. Upon examining the original spectra, we observe that there are no clear peaks increasing with CTB concentrations in these regions. Under such conditions, even when the intensity variation of the CTB-treated samples in these regions is small, the signals in these regions would not be emphasized during the decision-making process. This further supports the CNN model’s ability to extract peak features, as these features can reproducibly occur.

This is an encouraging outcome, as it indicates that the model not only recognizes the presence of peaks but also ensures that these peaks are consistently observed. Given that biological samples can sometimes contain impurities or be partially damaged by laser exposure, it is important to exclude peaks caused by such unstable factors as unreliable features. Consequently, the Grad-AM-generated saliency scores may better highlight stable characteristic peaks that are likely to appear under practical experimental conditions.

### Interpretable Multiscale Spectral Features Revealed by Grad-AM Saliency Score

**Figure 7:**
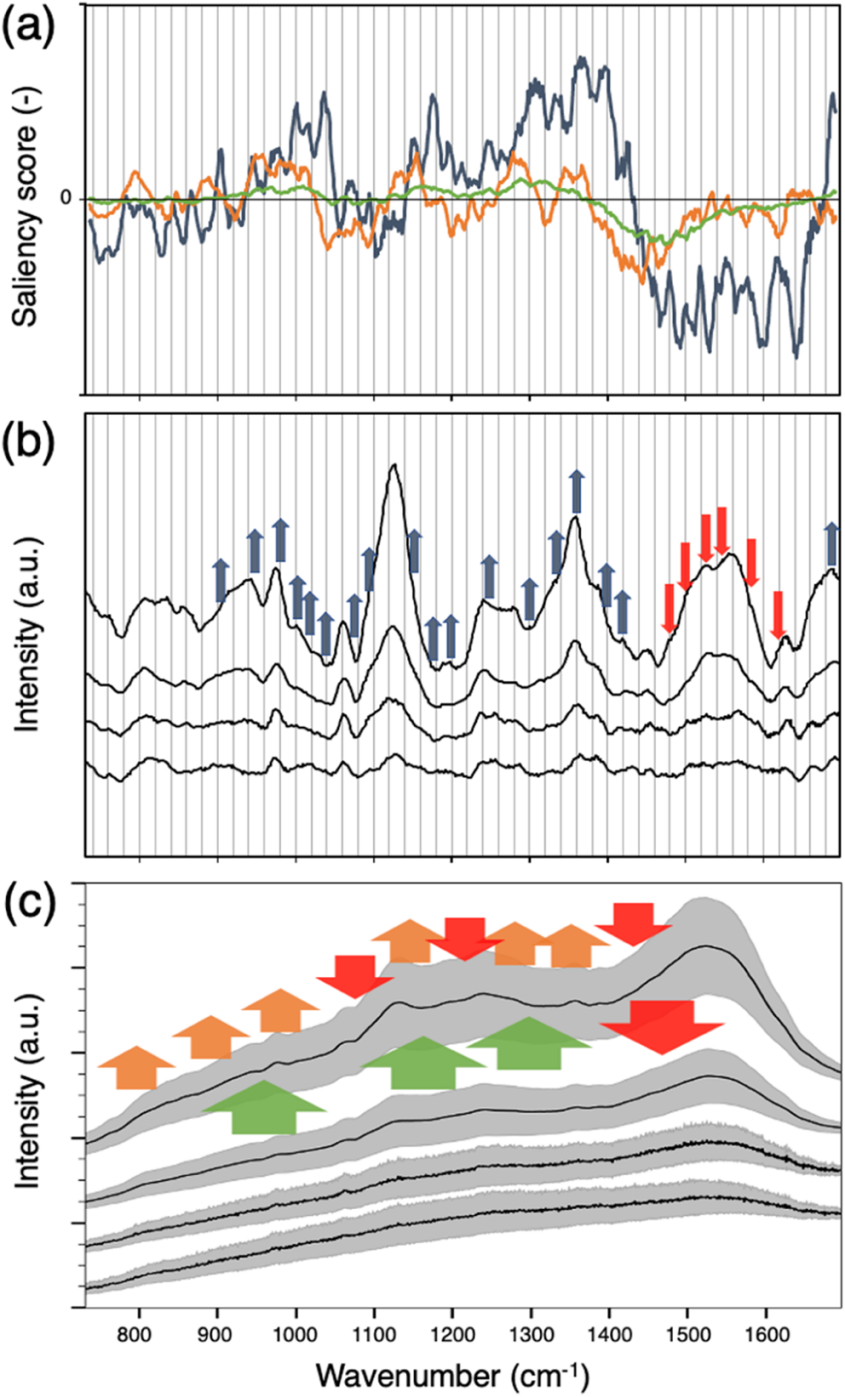
Grad-AM saliency scores and spectral feature analysis. (a) Saliency score contributions from different kernel sizes: 21 cm^–1^ (blue), 63 cm^–1^ (orange), and 152 cm^–1^ (green), highlighting spectral features at different scales. (b) Background-subtracted spectra with arrows marking regions where the 21 cm^–1^ kernel size saliency score shows positive (blue) and negative (red) contributions, corresponding to CTB concentration-dependent peaks. (c) Raw spectra with arrows highlighting regions where the saliency scores for kernel sizes of 63 and 152 cm^–1^ indicate positive contributions (orange and green) and negative contributions (red), corresponding to spectral variations. In (b, c), the spectra, from top to bottom, correspond to the samples treated with 100, 10, and 1 ng/mL CTB, followed by the non-CTB sample.

We analyzed the Grad-AM saliency score by separating the contributions from three different kernel sizes and plotted them individually in Figure 7a. The blue line represents the saliency score contributed by the kernel size of 21 cm^–1^, the orange line represents the contribution from the kernel size of 63 cm^–1^, and the green line represents the contribution from the kernel size of 152 cm^–1^. It is clear that the curves derived from each kernel size highlighted peaks or bands with widths corresponding approximately to the scale of their respective kernel sizes.

We aimed to determine whether these highlighted regions revealed by saliency score curves could correspond to the characteristic peaks of CTB. In this study, we used the CNN model to differentiate between non-CTB samples and CTB-treated samples exposed to 1 ng/mL CTB. As mentioned earlier, the original spectra of these two groups did not exhibit statistically significant differences at all wavenumbers. To identify the peaks generated by CTB treatment, we introduced spectra from higher concentrations for comparison to determine which peaks or bands increased with CTB concentration. Since the original spectra contained substantial background and noise, we performed background subtraction and smoothing across spectra of various concentrations to pinpoint the precise positions of the peaks, as shown in Figure 7b. We compared these identified peak positions with the regions highlighted by the blue curve in Figure 7a to evaluate whether the saliency score curves could highlight characteristic peaks of the target species or if the existence of peaks at these highlighted regions were indeed indicative of the presence of CTB.

We labeled the locations where the saliency score, derived from the 21 cm^–1^ kernel size, shows a positive contribution with blue arrows in the background-subtracted spectra in Figure 7b, while red arrows were used to denote regions with a negative contribution. Indeed, the peaks at the wavenumbers marked by the blue arrows exhibit an increase in intensity with increasing CTB concentration. Interestingly, at the wavenumbers indicated by the red arrows, the peaks also appear to increase with CTB concentration, but the saliency score map assigns a negative influence to these regions. As mentioned in the previous subsection, we think that this occurs because, in these regions, non-CTB samples exhibit smaller intensity variations compared to the 1 ng/mL CTB-treated samples, as shown in the spectrum standard deviation in Figure 7c for the 1450–1650 cm^–1^ region. This result again supports that the Grad-AM saliency score map not only identifies which peaks are prominent but also accounts for the variation in intensity across the data.

As for the orange and green lines in Figure 7a, which represent saliency scores contributed by the 63 and 152 cm^–1^ kernel sizes, the corresponding impacts are difficult to discern from the background-subtracted spectra. To better understand these contributions, we examined the raw spectra at different concentrations to observe how the regions highlighted by the orange line change with increasing CTB concentration. As shown in Figure 7c, orange arrows indicate regions where the saliency score from the 63 cm^–1^ kernel size makes a positive contribution, while red arrows mark regions with a negative contribution. Similarly, wide green arrows highlight regions where the 152 cm^–1^ kernel size contributes positively, whereas wide red arrows denote regions with a negative contribution. From the raw spectra in Figure 7c, it is evident that the wavenumber regions highlighted by the orange arrows exhibit an upward trend with increasing concentration. Conversely, in the regions marked by downward red arrows, any intensity increase tends to flatten the spectral curve, making neighboring peaks less distinct and more similar to the broader non-CTB spectral profile. The regions indicated by the wide green and red arrows appear to have similar effects, though they are less discernible to the naked eye.

Table 5 presents the locations and regions highlighted by the saliency score curves from the 21, and 63 cm^–1^ kernels, along with their potential correspondence to the peaks and bands of key amino acids and structural features in CTB. The ten most abundant amino acids in CTB are Ala, Ile, Lys, Thr, Leu, Ser, Asn, Glu, Phe, and Val (details in the SI). The locations highlighted by the saliency scores from the 21 cm^–1^ kernel size align well with the characteristic peaks of these amino acids. Additionally, several regions positively influenced by the orange line correspond to amino acid peak clusters. Meanwhile, the regions positively influenced by the green line generally correspond to those highlighted by the orange line and may also be associated with clusters of amino acid peaks.^56^

**Table 5:** Peaks and Bands Highlighted by Grad-AM Saliency Scores and Their Correspondence to the Key Amino Acids and Structural Features within CTB

peak/band	potential source	assignment	refs
903	Thr(903)	C–C–O in-phase stretch (secondary alcohols)	(56,63)
946	Leu(945), Val(947)	C–C stretching/CCN stretch/COO- stretching	(56,63−67)
980	Ser(976)	C–N stretching	(55,68)
1000	Phe(1002)	Symmetric ring breathing and vibration	(56,61,64,69−71)
1018	Ala(1015)	C–C stretch/CN stretch/C-NH_2_ stretch	(56,63−65,72)
1037	Ile(1033), Lys(1033)	CH_2_ wagging	(55,56,66,73)
1074	Lys(1076), Asn(1072)	C–C stretching/C–O stretching	(55,56,63,73,74)
1093	Ser(1089/1097)	C–O stretching	(55,68)
1148	Ala(1145)	C–C stretching/NH_2_ twist/NH_3_ wagging	(56,63,64,71,75,76)
1177	Glu(1176)	C–CN antisymmetric stretching	(56,63,64)
1196	Ile(1192), Thr(1194)	CH_2_ twist and rock/NH_3_ rocking/CH-NH_2_ stretching	(56,63,65)
1246	Leu(1242)	CH_2_ twist and rock	(56,63,66)
1296	Ser(1301), Glu(1301)	CH_2_ twist and rock/CH_2_ deformation	(56,63,74)
1332	Ile(1327), Asn(1330)	CH deformation/COO- stretching	(56,63−65)
1359	Ala(1358), Ile(1354), Lys(1360)	CH deformation/COO-stretching/CH_3_ deformation/CNH_3_ stretching	(55,56,63−65,71)
1397	Ile(1397), Val(1396)	COO- symmetric stretching/Cα2H2 deformation/C–N stretching	(56,63,71,72,77)
1419	Thr(1417), Asn(1415)	COO- symmetric stretching/Cα2H2 deformation/C–N stretching	(56,63,71,72,77)
1685	Amide I (β-sheet 1634–1697)		(69,78−80)
774–819	Ala(772), Ile(765/820), Thr(771), Asn(876), Val(776)	COO- bending/C–C skeletal stretching	(55,56,63−65)
860–915	Ile(853), Thr(871/903), Asn(876), Glu(866)	C–C skeletal stretching/C–CH_3_ stretching/C–C–N stretching/C–COO stretching	(56,63−65,71,72,76)
950–1005	Ile(992), Leu(962)	CH_2_ wagging	(55,56,66)
1118–1165	Ala(1145), Ile(1132), Lys(1143), Leu(1130), Asn(1158), Val(1125)	C–C stretching/NH_2_ twist/NH_3_ wagging/C–CN antisymmetric stretching	(55,56,63,64,71,72,76)
1256–1311	Ala(1304), Ile(1257/1259), Thr(1308), Ser(1301), Asn(1261), Glu(1301), amide III (α-helix 1265–1280)	CH_2_ twist and rock/C–N–H bending/CH_2_ deformation	(55,56,61,66,69,74,78)
1326–1381	Ala(1358), Ile(1327/1354), Lys(1360), Thr(1339/1341), Leu(1342/1345), Ser(1371), Asn(1330), Glu(1373), Phe(1365)	CH deformation/COO- stretching/CH deformation/CNH_3_ stretching	(55,56,63−65,71,74)

This result is prominent because the Grad-AM approach could identify these peaks using the 1 ng/mL CNN model, even though traditional statistical analysis could not discern such differences. While proper background subtraction and smoothing could also reveal some differences between non-CTB and 1 ng/mL CTB samples, the choice of background subtraction method often affects the relative size of the peaks and may sometimes remove broader bands as background. Figure 7b shows that, after performing 13th-order polynomial background subtraction on the raw spectral data, narrower peaks were well-preserved and aligned closely with the blue line (the saliency score derived from the 21 cm^–1^ kernel size). However, broader bands were partially removed. Thus, the impacts of the orange and green lines (saliency scores derived from the 63 and 152 cm^–1^ kernel sizes) are not easily observed in the background-subtracted spectral data but are better identified in the raw data.

Another notable observation is that certain individual kernels or specific combinations could also achieve high classification accuracy. This prompted us to examine the behavior of Grad-AM saliency scores in these cases. When analyzing the saliency scores of a single kernel, we found that the optimal kernels identified across five different levels highlighted similar spectral regions (Supporting Information, Figure S7). The saliency score curves as a function of wavenumber exhibited trends similar to the overall saliency score observed in our optimal multi-kernel combination (21, 63, and 152 cm^–1^). However, as kernel size increased, the curves gradually lost their ability to highlight smaller peaks. Interestingly, we observed the emergence of some distinct narrow peaks at levels D and E that did not correspond to the characteristic CTB peaks. We hypothesize that these peaks may arise from the difficulty larger kernels face in accurately capturing finer peak features, leading the model to produce oscillatory artifacts as a compensatory mechanism.

In contrast, when using optimal multi-kernel combination (21, 63, and 152 cm^–1^), the contribution of the score from level D (152 cm^–1^) did not exhibit pronounced oscillatory artifacts. This suggests that the presence of two smaller kernels enabled a more precise representation of narrow peaks, allowing level D to focus on its own scale without requiring compensatory adjustments. In addition, when analyzing the saliency score curve of a single kernel at level A, we observed that narrow peaks aggregated in order to compensate for the broader highlighted regions, slightly reducing the resolution of individual peaks. Taken together, these findings suggest that when classification-relevant peaks span multiple scales, assigning kernels to distinct feature sizes could enhance overall fitting and improve classification accuracy.

We also analyzed the Grad-AM saliency score curves for the 56, 81, and 218 cm^–1^ kernel combination (levels B, C, and E), as it also exhibited strong classification performance. As shown in Figure S8(a) in Supporting Information, this combination produced a well-fitted overall shape which possess similar positively- and negatively contributed regions. Despite this, it failed to highlight certain critical Raman peaks, such as the peak at 903 cm^–1^, which may correspond to the Raman signal of threonine (Thr), a major amino acid in CTB. This finding further supports the idea that using kernels with sizes comparable to actual Raman peaks enhances the detection of important spectral features.

## Conclusions

In this study, we investigated the challenge of classifying and analyzing SERS spectra of CTB binding on cell membranes. The low biomolecular concentrations and complex biological environments make it difficult to statistically differentiate treated cell membranes from nontreated ones. To address these challenges, we developed a multiscale 1D-CNN capable of extracting features at different scales through the careful analysis of convolutional kernel sizes. Our approach demonstrated superior performance compared to traditional machine learning methods and other multiscale CNNs in previous literature, achieving high accuracy, sensitivity, specificity, and precision. The optimal kernel size combination was identified through rigorous hyperparameter tuning, underscoring the importance of tailoring CNN architectures to the specific characteristics of the spectral data. Furthermore, the implementation of data augmentation significantly enhanced the model’s robustness and reduced the risk of overfitting, particularly in the context of limited biological data. Finally, we investigated the decision-making process of the CNN using three types of saliency heatmaps to visualize the critical spectral regions influencing the model’s predictions. Among these, the Grad-AM method we developed demonstrated superior performance, providing insights into how convolutional layers process complex biomolecular data. It effectively emphasizes key spectral features, including subtle differences that are difficult to distinguish through statistical analysis of the raw data. This underscores the potential of our visualization technique as a powerful tool for supporting classification accuracy and even identifying previously obscure peaks. Overall, our proposed multiscale 1D-CNN, combined with data augmentation, offers a powerful tool for the accurate classification and analysis of biomolecular Raman spectra, paving the way for more effective biomolecular identification in challenging environments.

## Supporting Information Available

Estimating the number of CTB molecules in the laser-illuminated area (Figure S1); structure of our proposed CNN (Figure S2); effect of convolutional kernel sizes on spectrum smoothing (Figure S3); gradient plots with the activation maps in the trained multiscale CNN model (Figure S4); saliency scores of the three visualization methods (Figure S5); smoothed saliency scores of the three visualization methods (Figure S6); comparison of Grad-AM saliency scores of single kernel CNN models (Figure S7); comparison of Grad-AM saliency scores of multiscale CNN models with strong classification performance (Figure S8); sequence of Cholera Toxin B subunit (CTB) and amino acid percentages (Table S1); tuned hyperparameters of the various kernel combinations for our proposed multiscale 1D-CNN with 5-fold cross-validation (Table S2); tuned hyperparameters for previous studies regarding multiscale 1D-CNNs with 5-fold cross-validation (Table S3); tuned hyperparameters for traditional machine learning algorithms with 5-fold cross-validation (Table S4) (PDF)

## Abbreviations

CNN: convolutional neural network
