# Supplementary material for: Interpretable Multiscale Convolutional Neural Network for Classification and Feature Visualization of Weak Raman Spectra of Biomolecules at Cell Membranes
Source: ACS Sens. 2025 Apr 4;10(4):2652–66. doi: 10.1021/acssensors.4c03260 (PMC12038881; doi:10.1021/acssensors.4c03260)
Supplement: Supplementary file 1 — se4c03260_si_001.pdf [file se4c03260_si_001.pdf]

Supporting Information

Interpretable Multiscale Convolutional Neural Network for Classification and Feature Visualization of Weak Raman Spectra of Biomolecules at Cell Membranes

Che-Lun Chin, Chia-En Chang, and Ling Chao*

Department of Chemical Engineering, National Taiwan University, No. 1, Sec. 4, Roosevelt Rd., Taipei 10617, Taiwan

*Correspondence should be addressed to Ling Chao (lingchao@ntu.edu.tw)

Table of contents:

1. Estimating the number of CTB molecules in the laser-illuminated area **(Figure S1)**	S2
2. Structure of our proposed CNN **(Figure S2)**	S3
3. Effect of convolutional kernel sizes on spectrum smoothing **(Figure S3)**	S4
4. Gradient plots with the activation maps in the trained multiscale CNN model (**Figure S4)**	S5
5. Saliency scores of the three visualization methods **(Figure S5)**	S12
6. Smoothed saliency scores of the three visualization methods **(Figure S6)**	S13
7. Comparison of Grad-AM saliency scores of single kernel CNN models **(Figure S7)**	S14
8. Comparison of Grad-AM saliency scores of multiscale CNN models with strong classification performance **(Figure S8)**	S15
9. Sequence of Cholera Toxin B subunit (CTB) and amino acid percentages **(Table S1)**	S16
10. Tuned hyperparameters of the various kernel combinations for our proposed multiscale 1D-CNN with 5-fold cross-validation **(Table S2)**	S17
11. Tuned hyperparameters for previous studies regarding multiscale 1D-CNNs with 5-fold cross-validation **(Table S3)**	S18
12. Tuned hyperparameters for traditional machine learning algorithms with 5-fold cross-validation **(Table S4)**	S18
References	S19

1. Estimating the number of CTB molecules in the laser-illuminated area

The diameter of our 633 nm laser beam is approximately 1 μm, corresponding to an illuminated area of about 785,000 nm^2. Assuming the projected area of a single lipid is ~0.724 nm^2 [1], this area contains approximately 1,084,250 lipids. Based on literature values [2, 3], the GM1 content in the cell membrane is approximately 0.05 mol%, corresponding to ~542 GM1 molecules in the illuminated area. If each GM1 binds a single CTB molecule, the maximum possible number of bound CTB molecules is 542. However, the actual number of bound CTB depends on the bulk CTB concentration.

To estimate the CTB binding under different bulk concentrations, we used fluorescently labeled CTB to measure the binding curve on our cell membrane platform. Figure S1 shows the measured fluorescence intensity at the membrane after treatment with varying bulk CTB concentrations. Assuming that all GM1 molecules are saturated with CTB at high concentrations, we estimated the bound CTB at different concentrations based on the fluorescence intensity ratio relative to saturation. At 1 ng/mL, the fluorescence intensity is approximately 9.2% of the saturated level, indicating that ~50 CTB molecules are bound in the illuminated area. At 10 ng/mL, the fluorescence intensity reaches ~38% of the saturation level, corresponding to ~208 bound CTB molecules. At 100 ng/mL, the fluorescence intensity increases to ~72% of the saturation level, indicating ~390 bound CTB molecules in the illuminated area. This estimation highlights the relatively low number of CTB molecules in the laser-illuminated area under our experimental conditions.

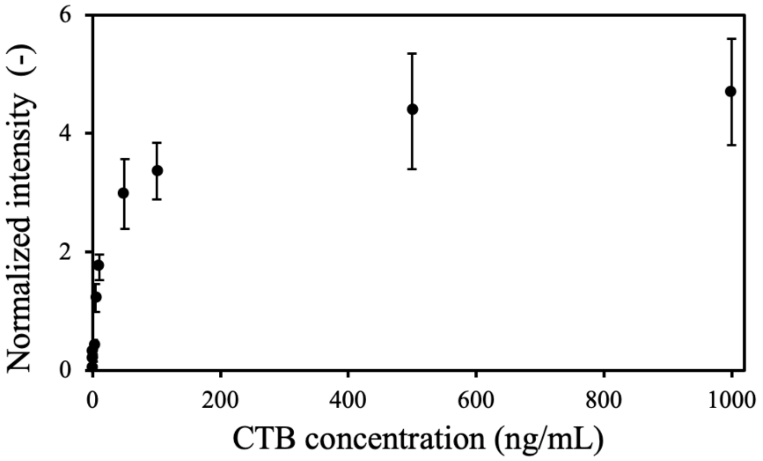

Figure S1. The binding curve of CTB molecules to the cell membrane platform, determined through fluorescence intensity measurements. The fluorescence intensity at the membrane was measured after treatment with varying bulk concentrations of fluorescently labeled CTB.

2. Structure of our proposed CNN

This figure illustrates a multiscale CNN with three parallel structures as an example. Each box represents a layer, with the corresponding input and output sizes indicated inside. The letter "k" represents the kernel size, while "ch" denotes the number of channels in the convolutional layers. A truncation layer is included in each parallel structure to ensure that the saliency heatmaps from different convolutional layers align properly. This ensures that the centers of the activation maps calculated for the saliency heatmaps are consistent across layers.

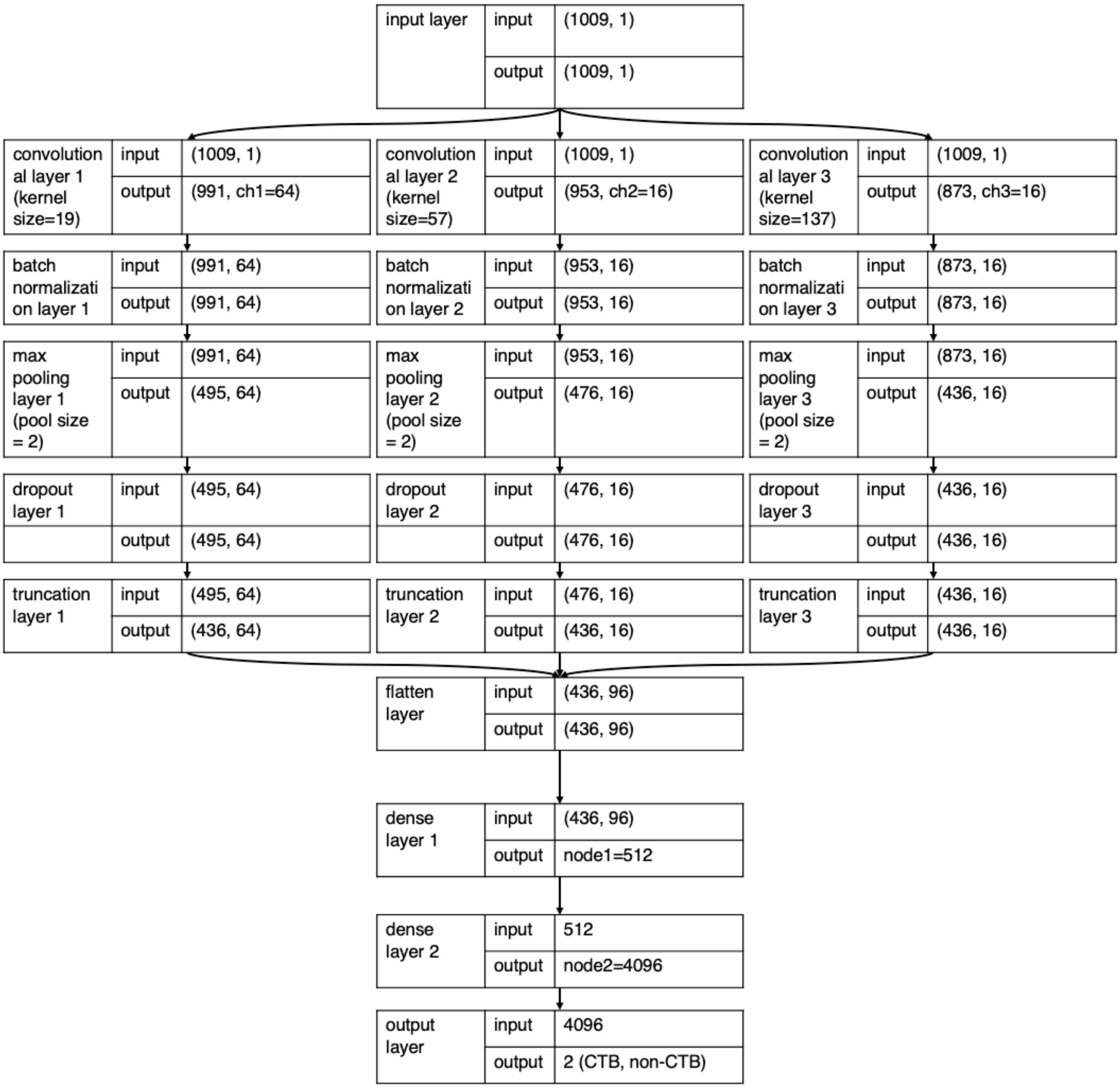

Figure S2. Structure of our proposed CNN. The input spectra are processed through three parallel 1D convolutional structures, each comprising convolution, batch normalization, max-pooling, and dropout layers. The features extracted from each parallel path are truncated, flattened, and then passed into fully connected layers, ultimately generating the final classification output. The calculation in the model is based on the actual spectral data points—intensity at each pixel of our spectrometer detector. In this study, a wavenumber range of 1117 cm^{-1} was recorded using 1009 pixels, resulting in a pixel width equivalent to 1.107 cm^{-1} per pixel.

3. Effect of convolutional kernel sizes on spectrum smoothing

To show how convolutional kernel sizes affect the smoothing of features in the spectrum of a 100 ng/mL CTB-treated sample, we applied convolutions using unit-value kernels of varying sizes. Figure S3 illustrates the progressive smoothing effect on spectral details. Smaller kernels preserve narrower features, while larger kernels smooth out finer details and emphasize broader trends. These results serve to demonstrate the trade-off in kernel size selection for balancing fine detail retention and broader feature extraction in CNN-based models.

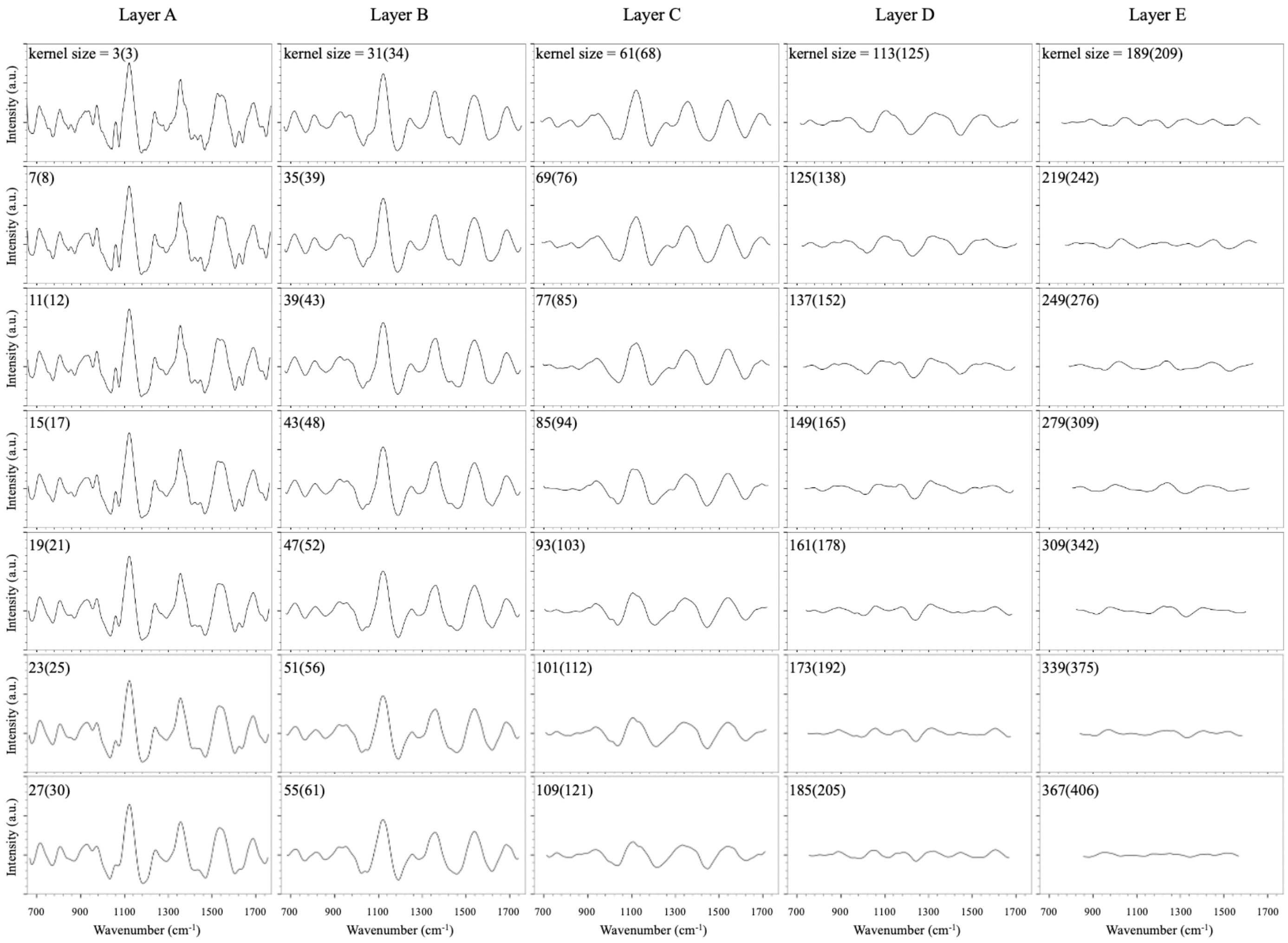

Figure S3. Activation maps of the SERS spectrum from a 100 ng/mL CTB-treated sample, generated by convolutional layers with unit-value kernels of varying sizes. Each map corresponds to a specific kernel size used for convolution. Smaller kernel sizes preserve fine details and capture narrow spectral features, while larger kernel sizes emphasize broader patterns and trends. The first value in each kernel size notation represents the pixel interval, while the value in parentheses indicates the corresponding size in wavenumbers. The spectrum shown is the average of all 22 spectra from 100 ng/mL CTB-treated samples, with background removal applied using a 13th-degree polynomial.

4. Gradient plots with the activation maps in the trained multiscale CNN model

In our model of best performance, the kernel number is 64 for kernels with a size of 19 (21 cm^{-1}), 16 for kernels with a size of 57 (63 cm^{-1}), and 16 for kernels with a size of 137 (152 cm^{-1}). The other hyperparameters are detailed in Table S2. With the mean of all spectra from test dataset as input spectra, we extracted the activation maps and the corresponding gradients of final output of CTB/non-CTB with respect to the values of activation maps.

(a)

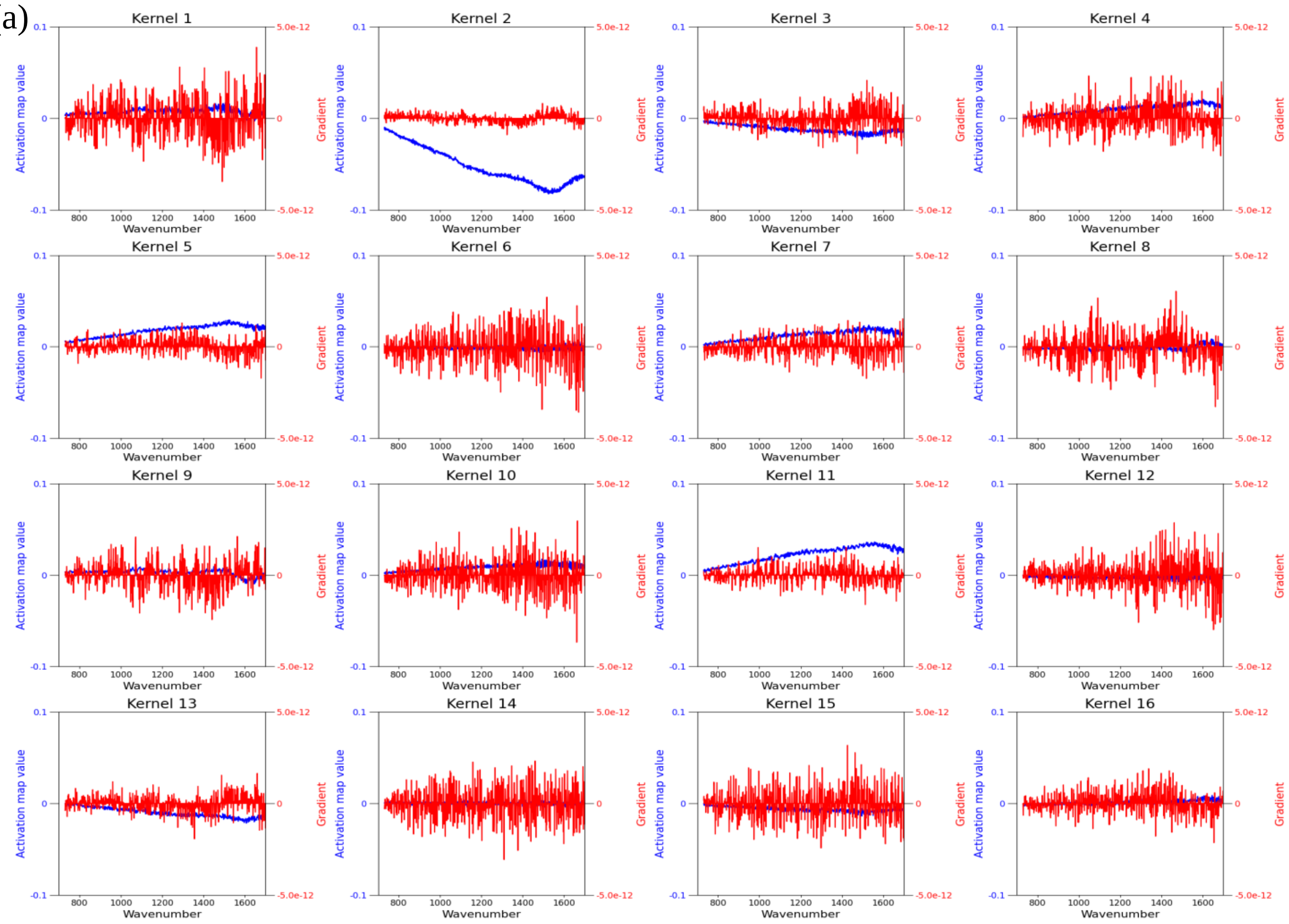

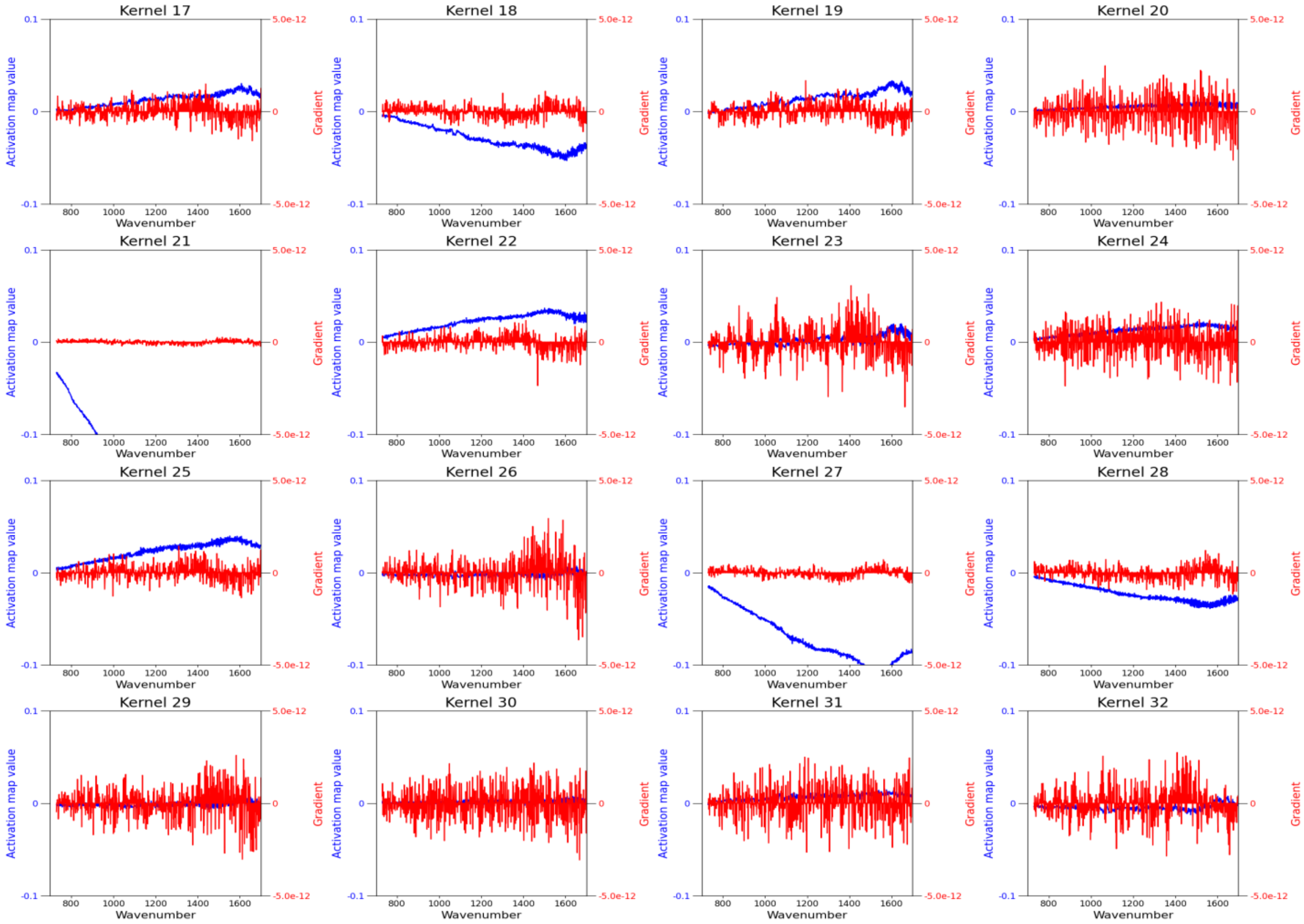
Kernel 17
Kernel 18
Kernel 19
Kernel 20
Kernel 21
Kernel 22
Kernel 23
Kernel 24
Kernel 25
Kernel 26
Kernel 27
Kernel 28
Kernel 29
Kernel 30
Kernel 31
Kernel 32
Activation map value
Gradient
Wavenumber
0.1
0
-0.1
5.0e-12
-5.0e-12
800
1000
1200
1400
1600

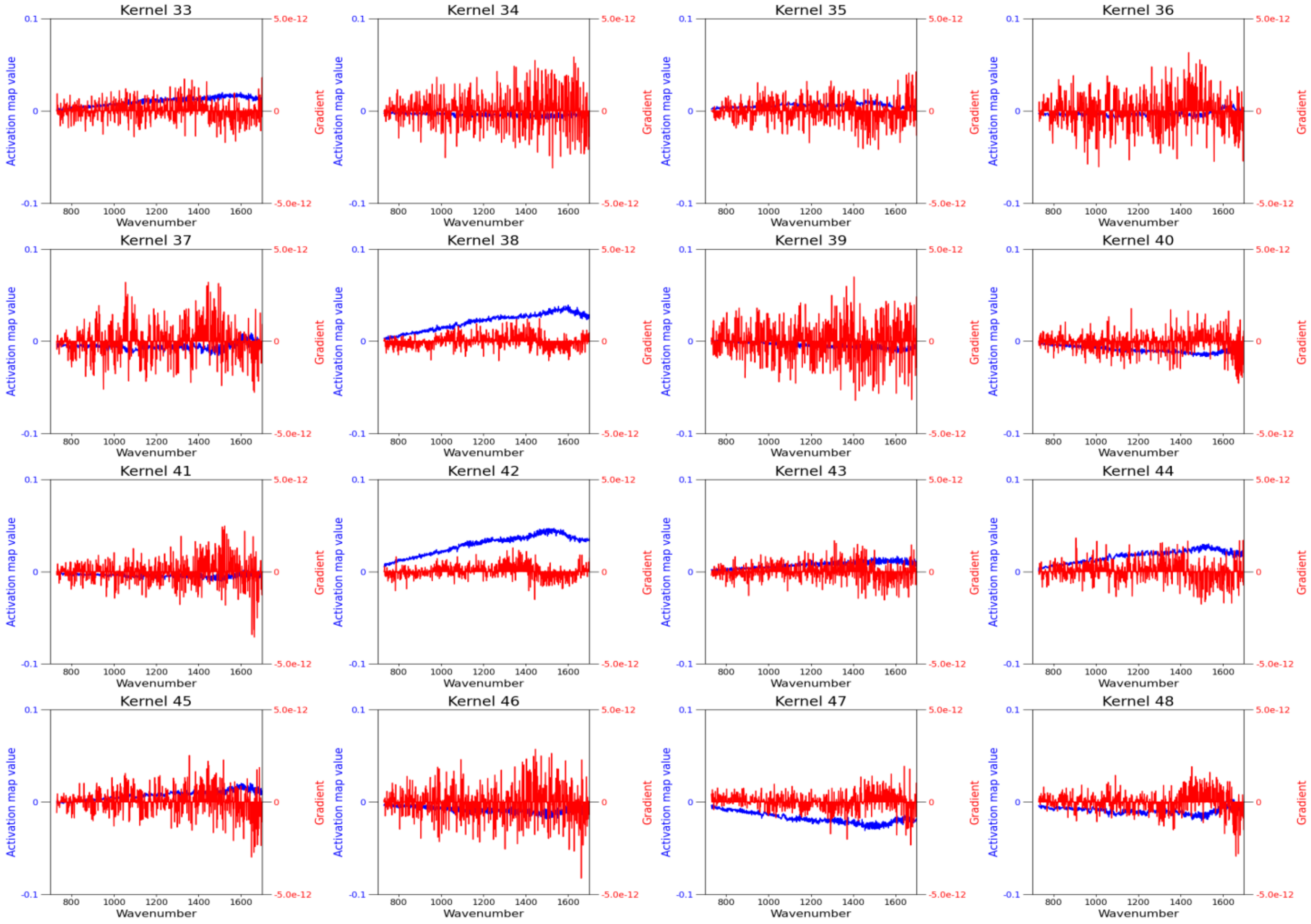
Kernel 33
Kernel 34
Kernel 35
Kernel 36
Kernel 37
Kernel 38
Kernel 39
Kernel 40
Kernel 41
Kernel 42
Kernel 43
Kernel 44
Kernel 45
Kernel 46
Kernel 47
Kernel 48
Activation map value
Gradient
Wavenumber
0.1
0
-0.1
5.0e-12
-5.0e-12
800
1000
1200
1400
1600

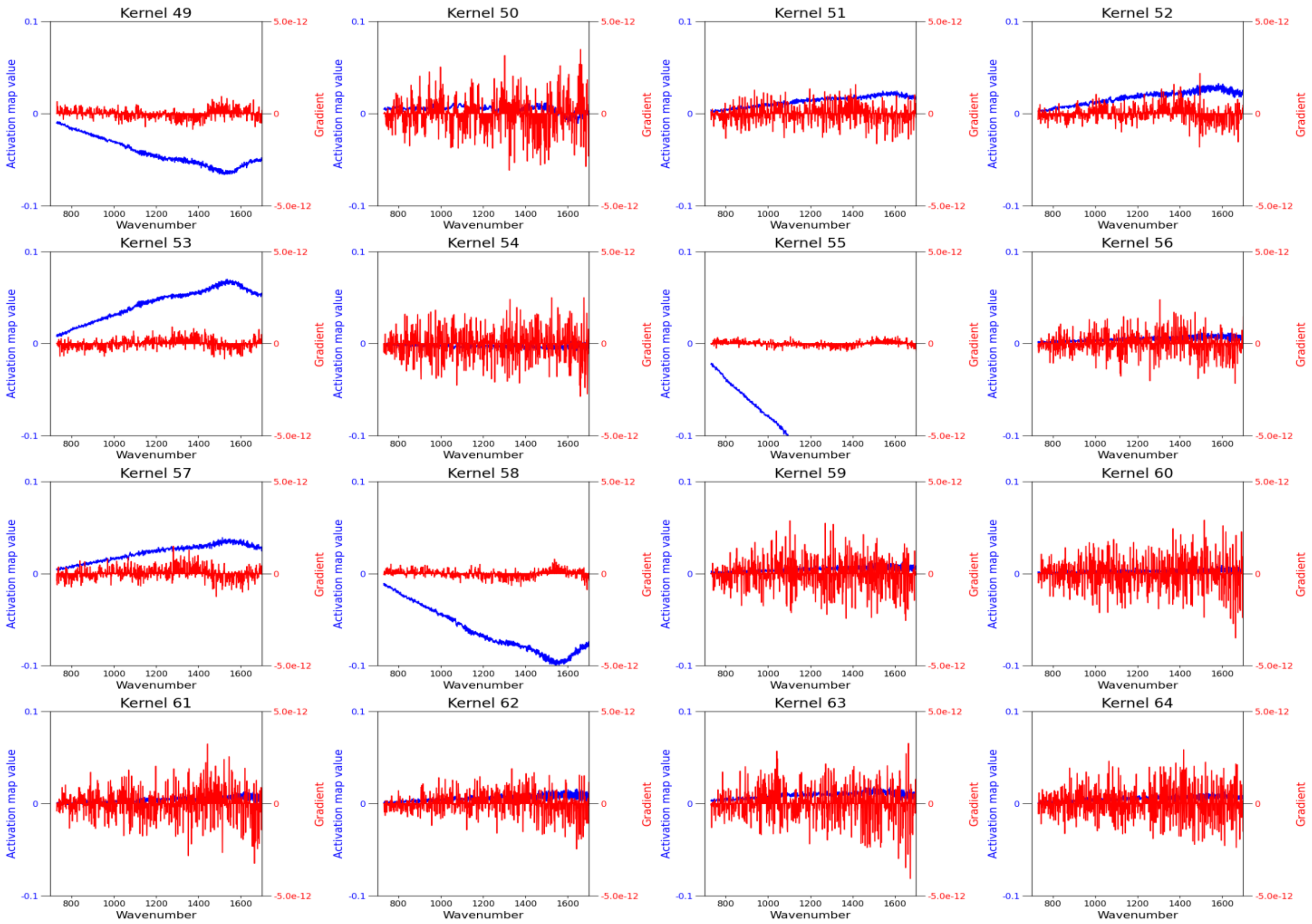
Kernel 49
Kernel 50
Kernel 51
Kernel 52
Kernel 53
Kernel 54
Kernel 55
Kernel 56
Kernel 57
Kernel 58
Kernel 59
Kernel 60
Kernel 61
Kernel 62
Kernel 63
Kernel 64
Activation map value
Gradient
Wavenumber
0.1
0
-0.1
5.0e-12
-5.0e-12
800
1000
1200
1400
1600

(b)

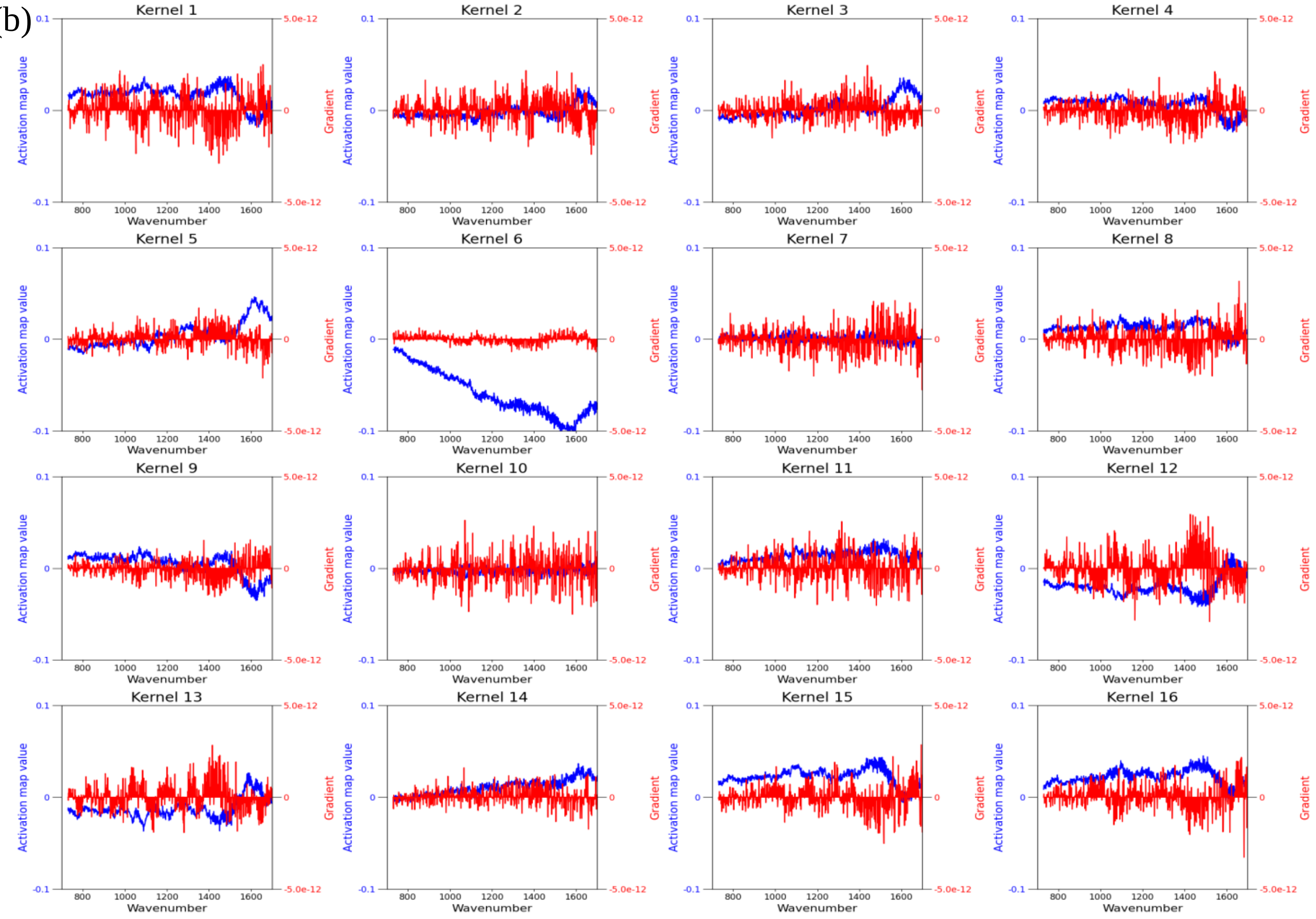
Kernel 1
Kernel 2
Kernel 3
Kernel 4
Kernel 5
Kernel 6
Kernel 7
Kernel 8
Kernel 9
Kernel 10
Kernel 11
Kernel 12
Kernel 13
Kernel 14
Kernel 15
Kernel 16
Activation map value
Gradient
Wavenumber
0.1
0
-0.1
5.0e-12
-5.0e-12
800
1000
1200
1400
1600

(c)

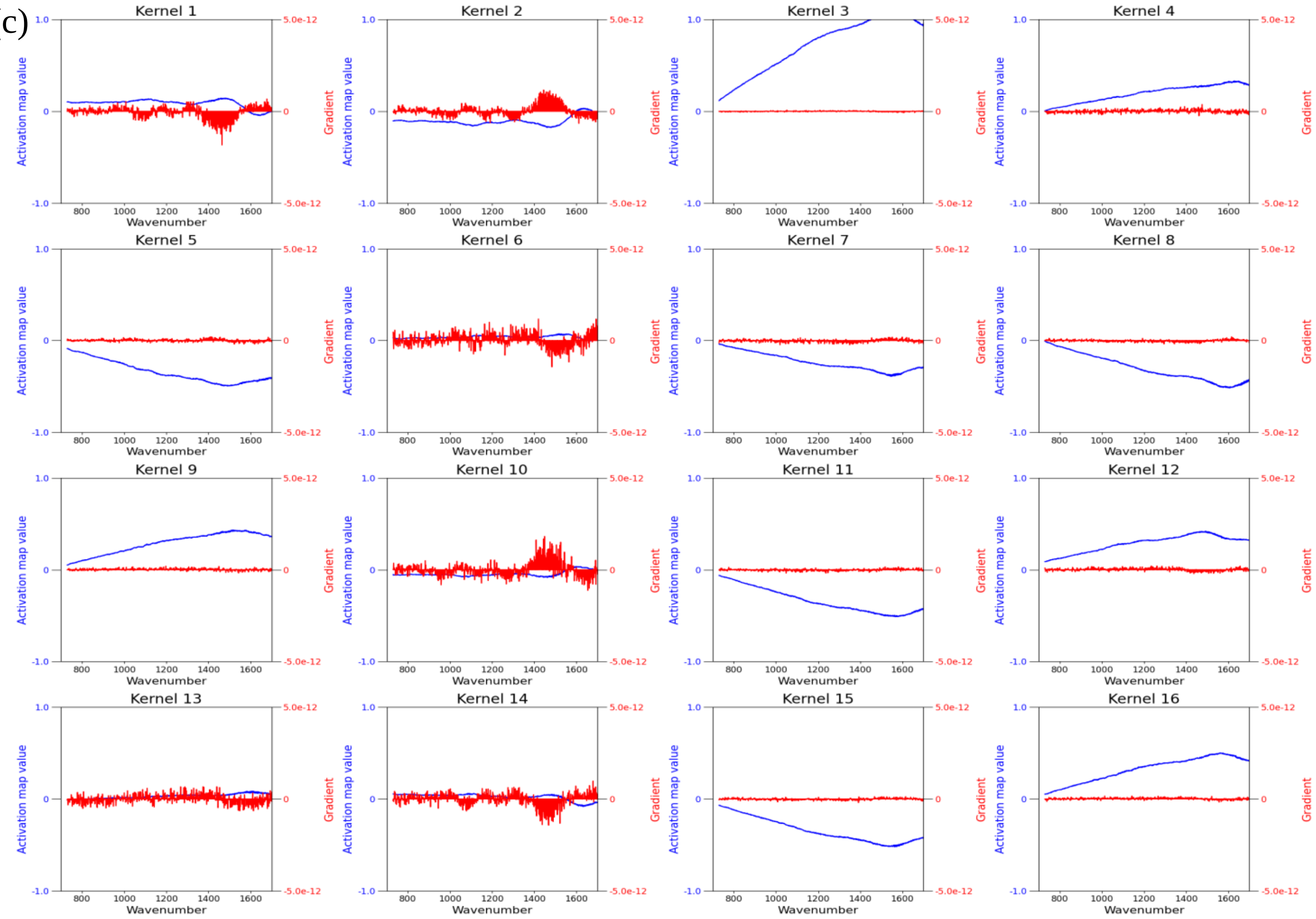
Kernel 1
Kernel 2
Kernel 3
Kernel 4
Kernel 5
Kernel 6
Kernel 7
Kernel 8
Kernel 9
Kernel 10
Kernel 11
Kernel 12
Kernel 13
Kernel 14
Kernel 15
Kernel 16
Activation map value
Gradient
Wavenumber
1.0
0
-1.0
5.0e-12
-5.0e-12
800
1000
1200
1400
1600

(d)

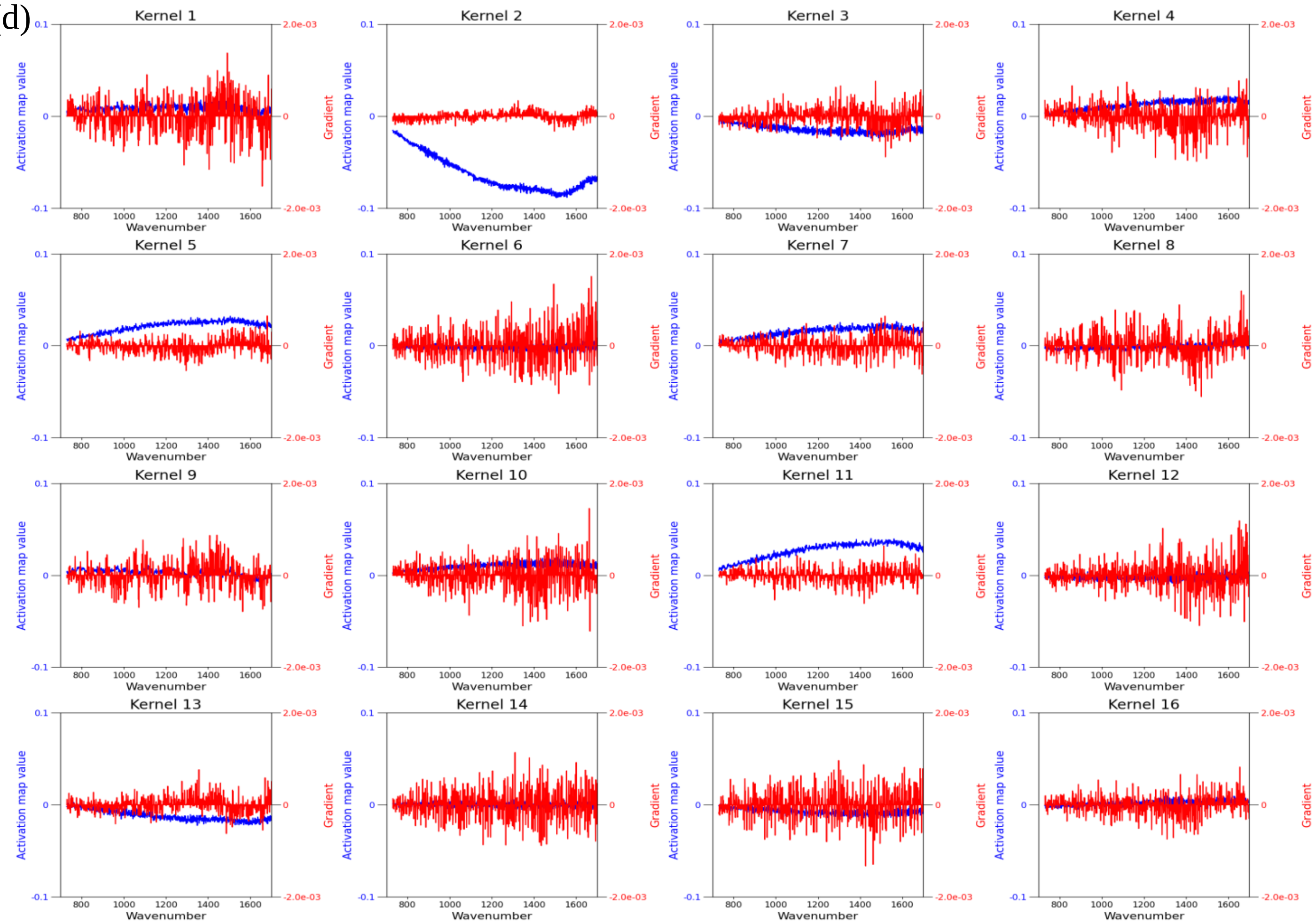
Kernel 1
Kernel 2
Kernel 3
Kernel 4
Kernel 5
Kernel 6
Kernel 7
Kernel 8
Kernel 9
Kernel 10
Kernel 11
Kernel 12
Kernel 13
Kernel 14
Kernel 15
Kernel 16
Activation map value
Gradient
Wavenumber
0.1
0
-0.1
2.0e-03
-2.0e-03
800
1000
1200
1400
1600

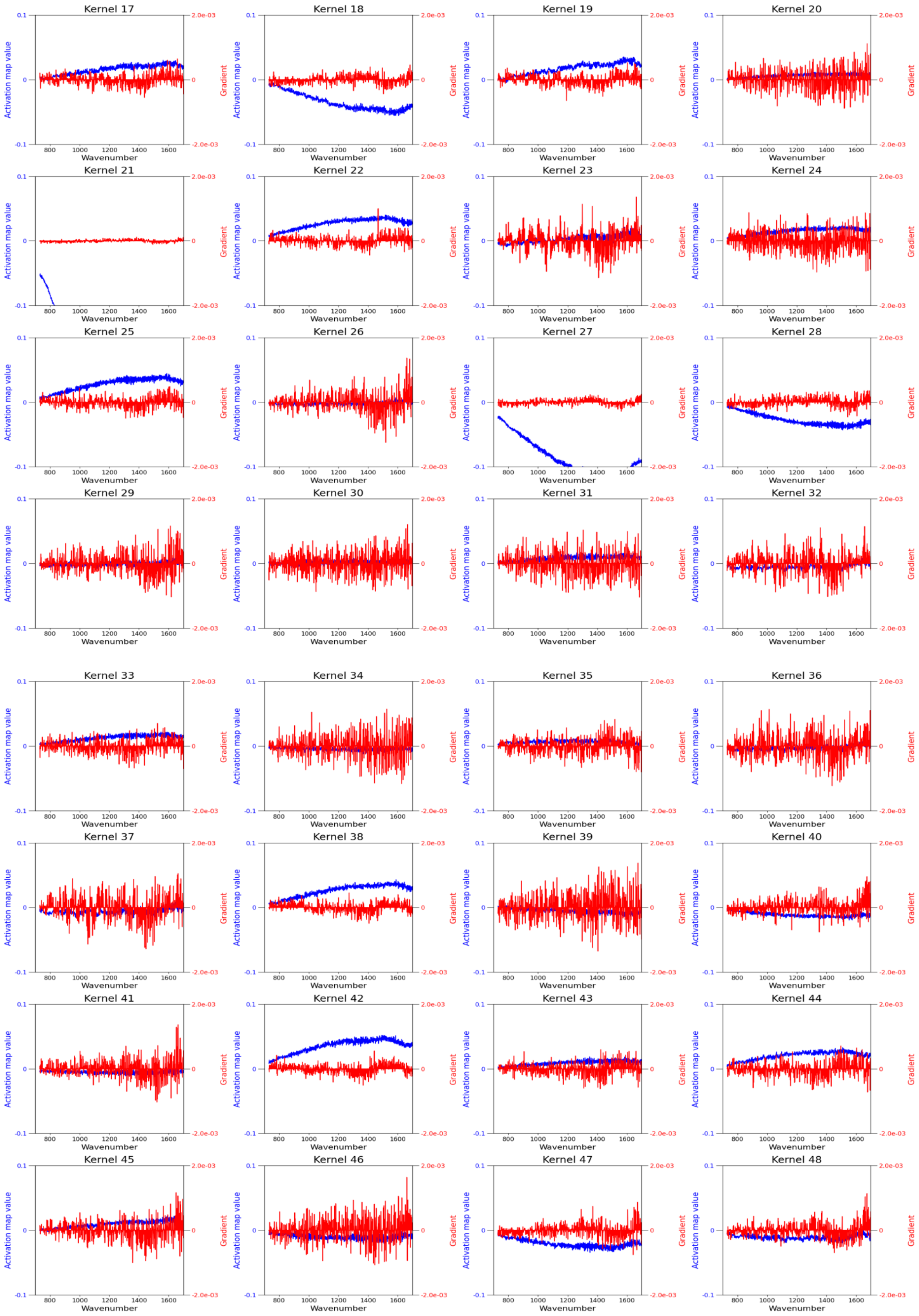
Kernel 17
Kernel 18
Kernel 19
Kernel 20
Kernel 21
Kernel 22
Kernel 23
Kernel 24
Kernel 25
Kernel 26
Kernel 27
Kernel 28
Kernel 29
Kernel 30
Kernel 31
Kernel 32
Kernel 33
Kernel 34
Kernel 35
Kernel 36
Kernel 37
Kernel 38
Kernel 39
Kernel 40
Kernel 41
Kernel 42
Kernel 43
Kernel 44
Kernel 45
Kernel 46
Kernel 47
Kernel 48
Activation map value
Gradient
Wavenumber
0.1
0
-0.1
2.0e-03
-2.0e-03
800
1000
1200
1400
1600

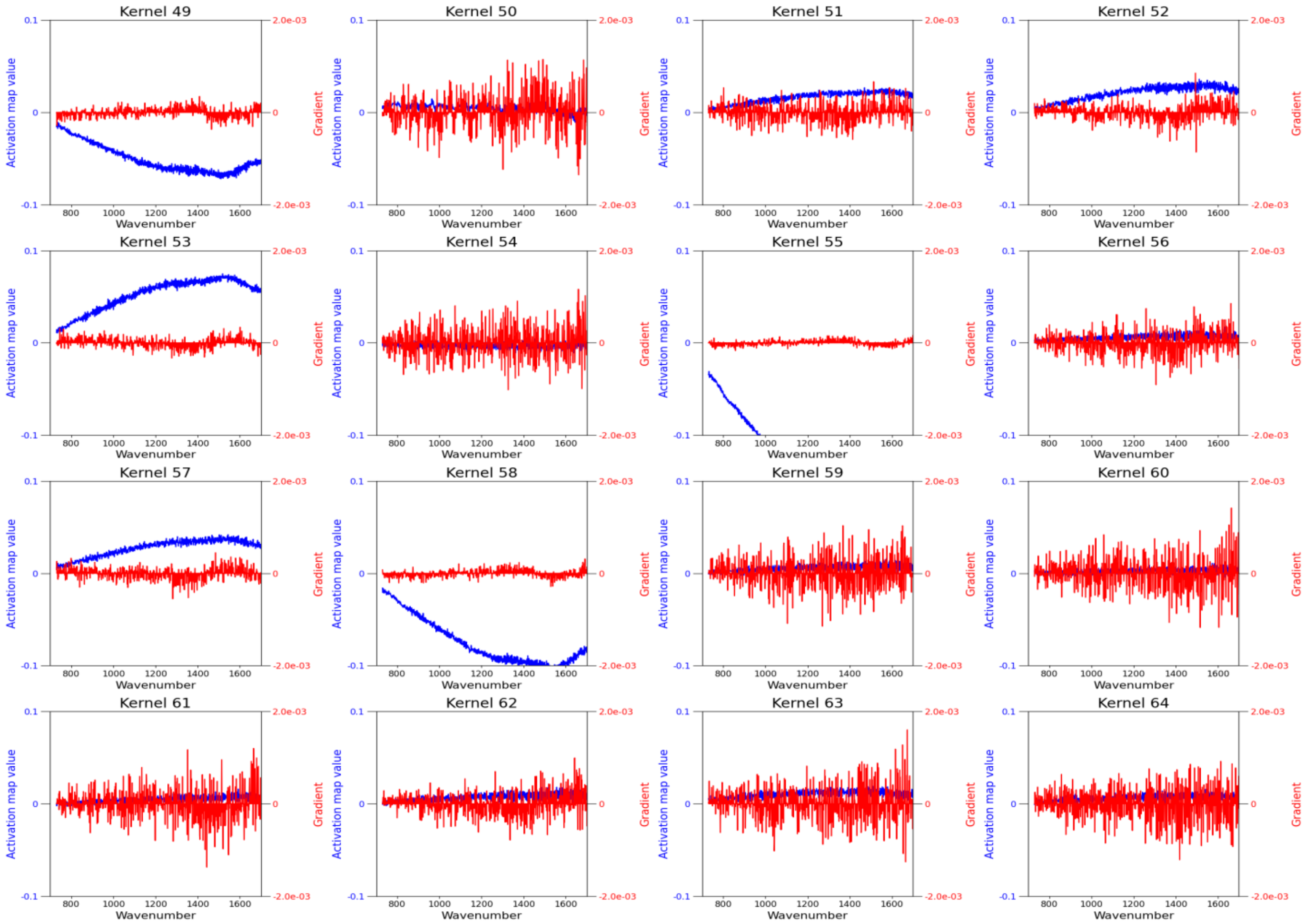
Kernel 49
Kernel 50
Kernel 51
Kernel 52
Kernel 53
Kernel 54
Kernel 55
Kernel 56
Kernel 57
Kernel 58
Kernel 59
Kernel 60
Kernel 61
Kernel 62
Kernel 63
Kernel 64
Activation map value
Gradient
Wavenumber
0.1
0
-0.1
2.0e-03
-2.0e-03
800
1000
1200
1400
1600

(e)

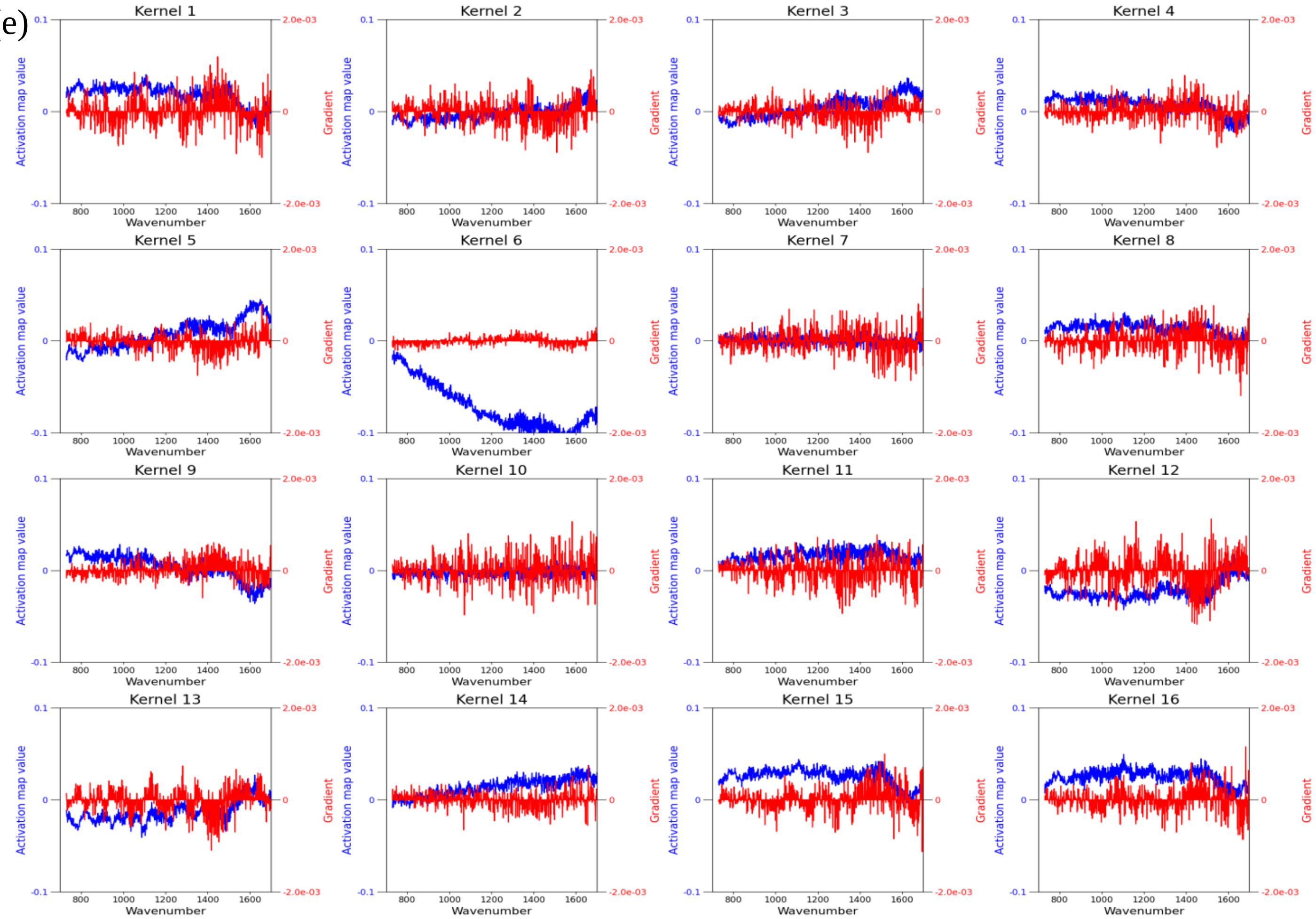
Kernel 1
Kernel 2
Kernel 3
Kernel 4
Kernel 5
Kernel 6
Kernel 7
Kernel 8
Kernel 9
Kernel 10
Kernel 11
Kernel 12
Kernel 13
Kernel 14
Kernel 15
Kernel 16
Activation map value
Gradient
Wavenumber
0.1
0
-0.1
2.0e-03
-2.0e-03
800
1000
1200
1400
1600

(f)

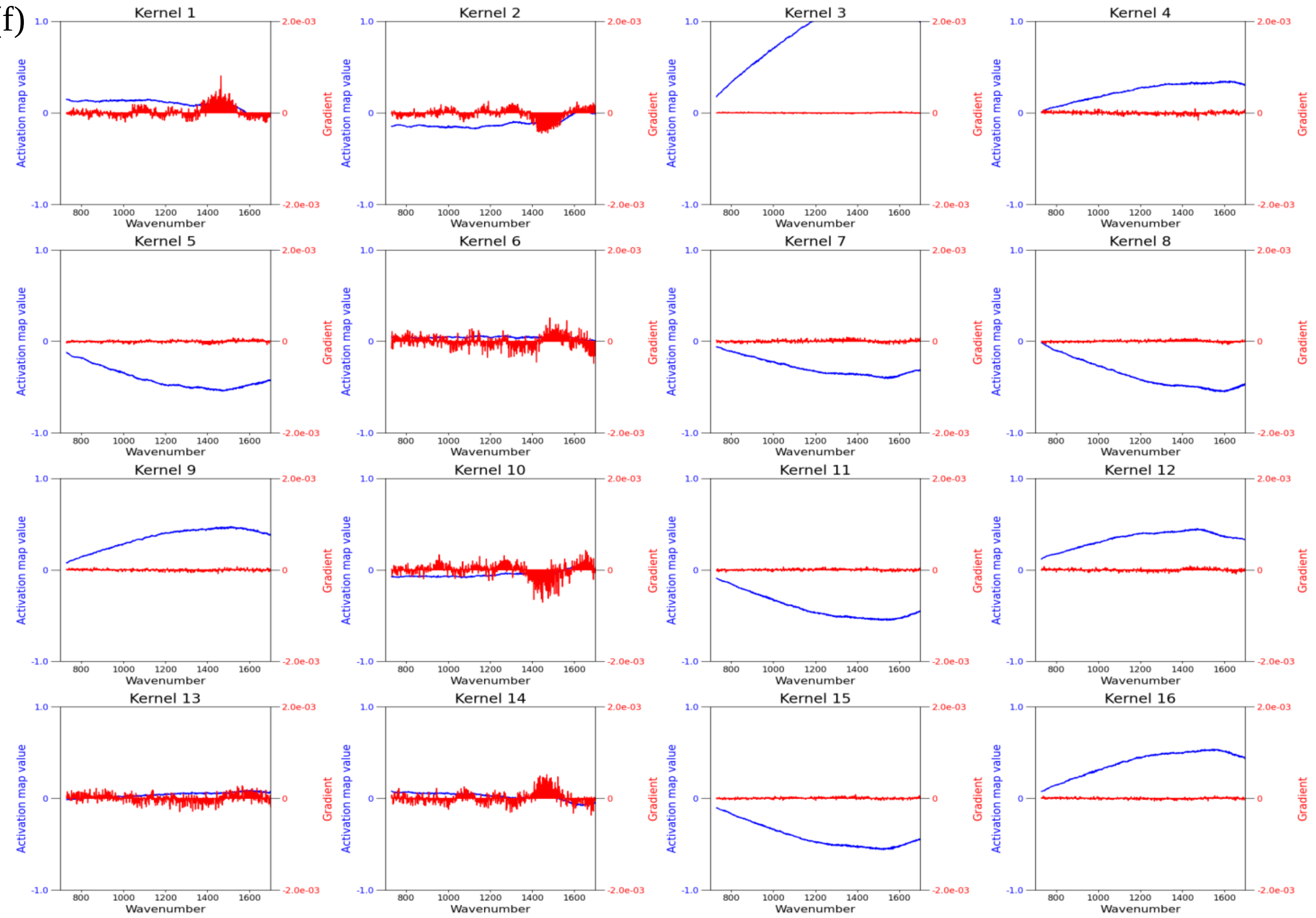

Figure S4. Activation maps (blue color) and the gradients of the final output for 1ng/mL CTB-treated and non-CTB samples with respect to their activation maps (red color). For CTB: (a) Kernels 1 to 64 for kernel size 19 (21 cm^{-1}), (b) Kernels 1 to 16 for kernel size 57 (63 cm^{-1}), and (c) Kernels 1 to 16 for kernel size 137 (152 cm^{-1}). For non-CTB: (d) Kernels 1 to 64 for kernel size 19 (21 cm^{-1}), (e) Kernels 1 to 16 for kernel size 57 (63 cm^{-1}), and (f) Kernels 1 to 16 for kernel size 137 (152 cm^{-1}).

5. **Saliency scores of the three visualization methods**

We calculated saliency scores of the three different visualization methods (Grad-AM; Grad-input; Grad-CAM) for spectra treated with three different CTB concentrations. The model was trained on spectra from 1 ng/mL CTB-treated and untreated samples, with equal instances of each. For Grad-AM and Grad-CAM, saliency scores were further decomposed to show contributions from the three kernel sizes in our CNN model.

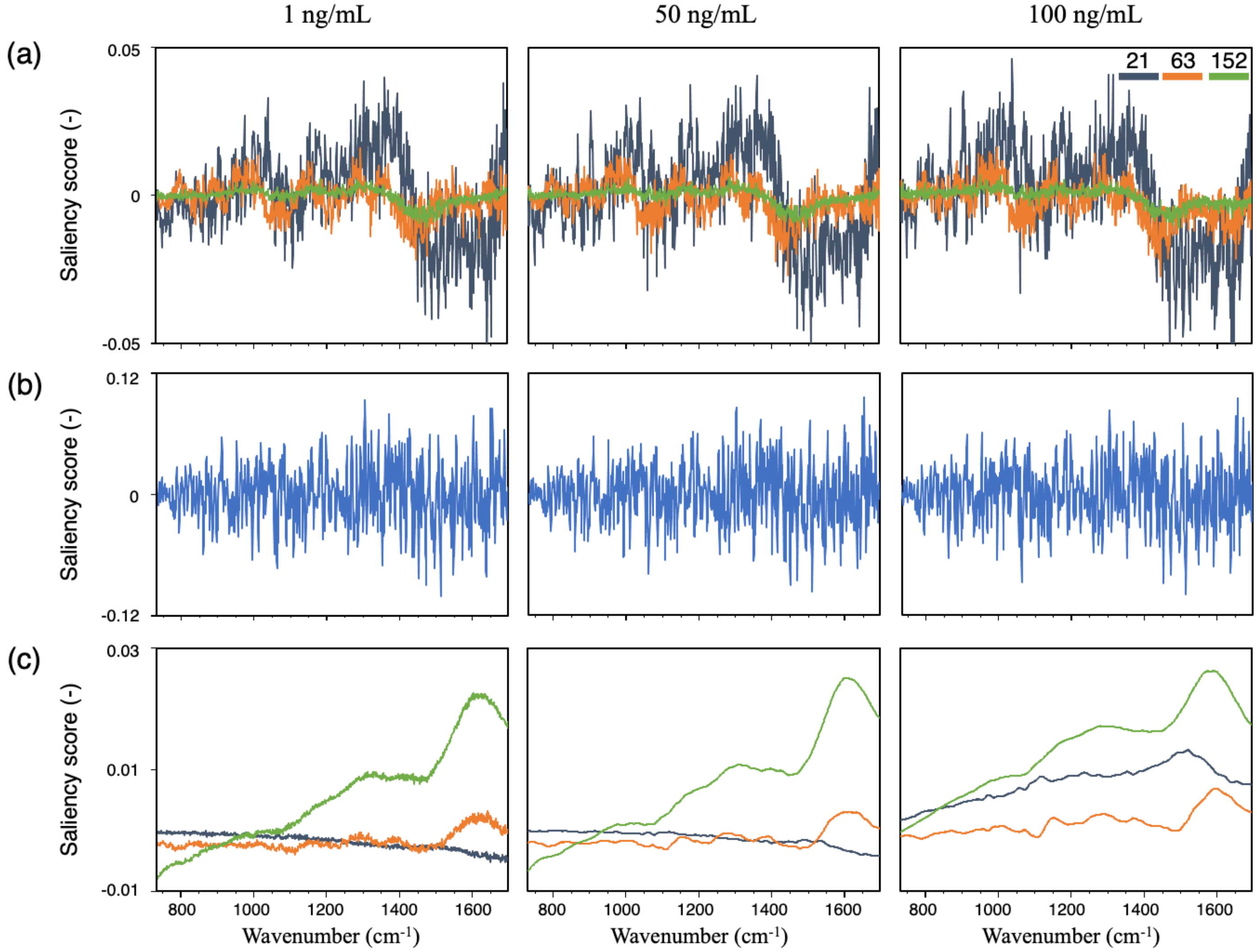

Figure S5. Saliency scores for (a) Grad-AM, (b) Grad-input, and (c) Grad-CAM. For Grad-AM and Grad-CAM, scores corresponding to kernel sizes of 21 cm^{-1}, 63 cm^{-1}, and 152 cm^{-1} are shown in gray, orange, and green, respectively. For Grad-input, the saliency score is obtained by calculating the gradient of the output with respect to the signal intensity in the input spectrum, and therefore no individual contribution from each of the three kernels can be obtained. Saliency scores were computed as the summation of normalized scores from each test spectrum, without data augmentation. Each test spectrum's saliency score was normalized by the sum of scores across different wavenumbers.

6. Smoothed saliency scores of the three visualization methods

To enhance the clarity of the saliency score trends, a moving average smoothing was applied with a window size of 11 pixels. The smoothed scores reveal more defined trends and better consistency across the concentrations compared to the unsmoothed results.

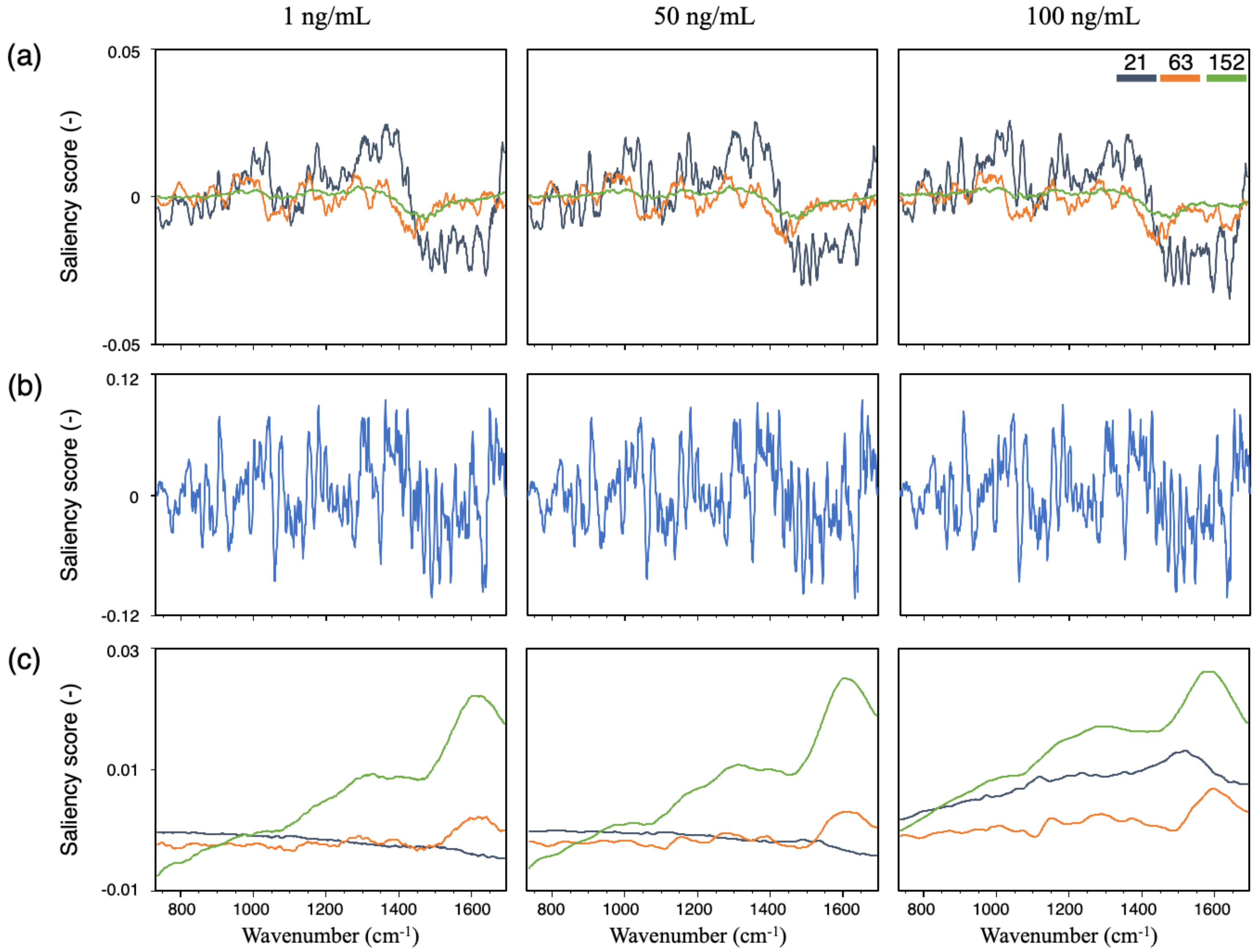

Figure S6. Smoothed saliency scores for (a) Grad-AM, (b) Grad-input, and (c) Grad-CAM. For Grad-AM and Grad-CAM, scores corresponding to kernel sizes of 21 cm^{-1}, 63 cm^{-1}, and 152 cm^{-1} are shown in gray, orange, and green, respectively. For Grad-input, the saliency score is obtained by calculating the gradient of the output with respect to the signal intensity in the input spectrum, and therefore no individual contribution from each of the three kernels can be obtained. Saliency scores were computed as the summation of normalized scores from each test spectrum, without data augmentation. Each test spectrum's saliency score was normalized by the sum of scores across different wavenumbers. A moving average with a window size of 11 pixels was applied for smoothing.

7. Comparison of Grad-AM saliency scores of single kernel CNN models

Figure S7(a) presents the Grad-AM saliency scores for single-kernel CNNs with kernel sizes of 23, 61, 90, 152, and 231 cm^{-1}. The saliency score curves across the five different kernel sizes show the similar pattern to the overall saliency distribution observed in our optimal multi-kernel combination (21, 63, and 152 cm^{-1}). However, as the kernel size increased, the ability to emphasize smaller peaks diminished. Although there are narrow peaks in levels D and E, we believe they are oscillatory artifacts. Many of them did not correspond to the CTB characteristic peaks that increase with concentration marked with blue arrows in Figure S7(b). This suggests that larger kernels have difficulty capturing fine spectral features, causing the model to generate these oscillations as a compensatory effect. On the other hand, at level A (23 cm^{-1}), narrow peaks tended to cluster together, compensating for the broader highlighted areas and slightly decreasing the resolution of distinct individual peaks.

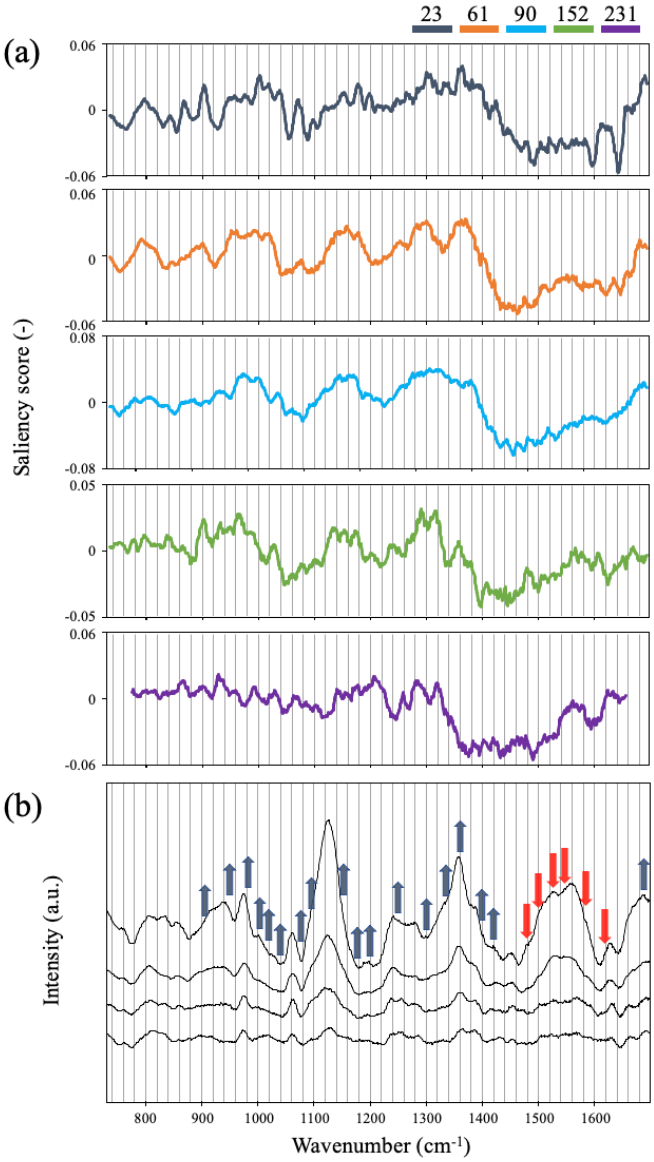

Figure S7. (a) Smoothed Grad-AM saliency scores of single kernel size CNNs with kernel sizes 23, 61, 90, 152 and 231 cm^{-1}, respectively. Scores corresponding to kernel sizes of 23 cm^{-1}, 61 cm^{-1}, 90 cm^{-1}, 152 cm^{-1}, and 231 cm^{-1} are shown in gray, orange, blue, green and violet, respectively. The scores were normalized by the sum of absolute values within each spectrum and then summed across the 21 original test spectra. A moving average smoothing was applied with a window size of 11 pixels. (b) Background-subtracted spectra with arrows marking regions where the 21 cm^{-1} kernel size saliency score shows positive (blue) and negative (red) contributions, corresponding to CTB concentration-dependent peaks. From top to bottom, correspond to the samples treated with 100, 10, and 1 ng/mL CTB, followed by the non-CTB sample.

8. Comparison of Grad-AM saliency scores of multiscale CNN models with strong classification performance

Figure S8(a) shows the Grad-AM saliency scores for a different kernel combination (56 cm⁻¹, 81 cm⁻¹, and 218 cm⁻¹) from a multi-scale CNN model with strong classification performance. While this combination produced an overall saliency score shape similar to that of our optimal kernel combination, with comparable positively and negatively contributed regions, it failed to highlight certain critical Raman peaks, such as the peak at 903 cm⁻¹. This peak may correspond to the Raman signal of threonine (Thr), a major amino acid in CTB. This comparison further support the idea that using kernel sizes comparable to actual Raman peaks enhances the detection of important spectral features.

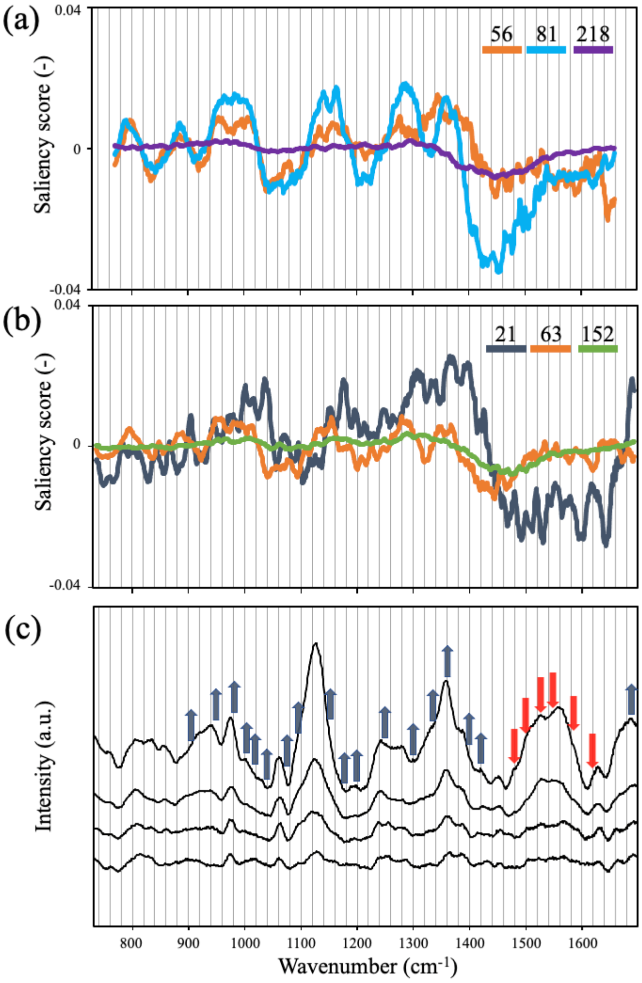

Figure S8. (a) Smoothed Grad-AM saliency scores of CNN with kernel sizes 56, 81, and 218 cm⁻¹. Scores corresponding to kernel sizes of 56 cm⁻¹, 81 cm⁻¹, and 218 cm⁻¹ are shown in orange, blue, and violet, respectively. (b) Smoothed Grad-AM saliency scores of CNN with kernel sizes 21, 63, and 152 cm⁻¹. Scores corresponding to kernel sizes of 21 cm⁻¹, 63 cm⁻¹, and 152 cm⁻¹ are shown in gray, orange, and green respectively. In (a) and (b), a moving average smoothing was applied with a window size of 11 pixels. The scores were normalized by the sum of absolute values within each spectrum and then summed across the 21 original test spectra. (c) Background-subtracted spectra with arrows marking regions where the 21 cm⁻¹ kernel size saliency score shows positive (blue) and negative (red) contributions, corresponding to CTB concentration-dependent peaks. From top to bottom, correspond to the samples treated with 100, 10, and 1 ng/mL CTB, followed by the non-CTB sample.

9. Sequence of Cholera Toxin B subunit (CTB) and amino acid percentages

The length of the sequence of Cholera Toxin B subunit is 124 residues and the sequence is as following,

MIKLKFGVFFTVLLSSAYAHGTPQNITDLCAEYHNTQIYTLNDKIFSYTESLAGKREMAIIT FKNGAIFQVEVPGSQHIDSQKKAIERMKDTLRIAYLTEAKVEKLCVWNNKTPHAIAAISMAN.

Table S1. Amino acid percentages of CTB.

Amino acid	Ratio
Ala	10.5%
Ile	9.7%
Lys	8.9%
Thr	8.1%
Leu	7.3%
Ser	5.6%
Asn	5.6%
Glu	5.6%
Phe	4.8%
Val	4.8%
Gly	4.0%
Tyr	4.0%
Gln	4.0%
Met	3.2%
His	3.2%
Asp	3.2%
Pro	2.4%
Arg	2.4%
Cys	1.6%
Trp	0.8%

Table S2. Tuned hyperparameters of the various kernel combinations for our proposed multiscale 1D-CNN with 5-fold cross-validation. Here, we list only the best hyperparameter and kernel combination identified for each type of layer combination.

Layer combination	Kernel size combination (pixel interval)	Kernel size combination (cm^{-1})	Channel	Dropout rate	Node	Learning rate
A	21	23	64	0.3	2048, 2048	2.0×10^{-4}
B	55	61	32	0.4	32, 1024	3.9×10^{-4}
C	81	90	32	0.3	2048, 2048	2.9×10^{-4}
D	137	152	16	0.1	2048, 2048	1.7×10^{-4}
E	209	231	16	0.1	2048, 256	9.5×10^{-5}
AB	9, 47	10, 52	64, 32	0, 0.2	1024, 128	1.5×10^{-4}
AC	23, 69	25, 76	16, 16	0.2, 0.1	128, 4096	2.4×10^{-4}
AD	13, 115	14, 127	16, 16	0, 0	128, 4096	1.4×10^{-4}
AE	17, 341	19, 378	32, 16	0.1, 0	2048, 2048	2.2×10^{-4}
BC	45, 71	50, 79	32, 16	0, 0	128, 2048	3.2×10^{-4}
BD	53, 155	59, 172	32, 128	0.1, 0.1	32, 256	1.0×10^{-4}
BE	41, 321	45, 355	16, 16	0.2, 0.1	128, 4096	5.1×10^{-4}
CD	61, 171	68, 189	16, 16	0.2, 0.1	512, 4096	3.3×10^{-4}
CE	69, 213	76, 236	16, 16	0.2, 0.1	2048, 4096	2.4×10^{-4}
DE	167, 237	185, 262	16, 64	0, 0.2	128, 512	2.4×10^{-5}
ABC	11, 33, 89	12, 37, 99	32, 32, 16	0.1, 0.3, 0.4	64, 1024	2.4×10^{-4}
ABD	19, 57, 137	21, 63, 152	64, 16, 16	0.3, 0.1, 0.4	512, 4096	3.3×10^{-4}
ABE	27, 33, 233	30, 37, 258	32, 32, 16	0, 0.3, 0.4	256, 1024	4.4×10^{-4}
ACD	23, 77, 139	25, 85, 154	32, 32, 16	0.5, 0.3, 0.4	16, 2048	6.0×10^{-4}
ACE	27, 71, 253	30, 79, 280	32, 16, 16	0.1, 0.1, 0.4	1024, 128	1.1×10^{-4}
ADE	23, 157, 225	25, 174, 249	64, 16, 128	0.2, 0, 0.4	128, 2048	3.7×10^{-4}
BCD	45, 107, 171	50, 118, 189	16, 128, 16	0.1, 0.4, 0.5	128, 4096	1.7×10^{-4}
BCE	51, 73, 197	56, 81, 218	32, 32, 16	0, 0.3, 0.4	1024, 64	3.5×10^{-4}
BDE	51, 137, 231	56, 152, 256	64, 16, 16	0.3, 0.5, 0.4	512, 4096	1.7×10^{-4}
CDE	103, 137, 295	114, 152, 327	32, 16, 16	0.1, 0.1, 0.4	512, 4096	1.9×10^{-4}
ABCD	21, 47, 69, 133	23, 52, 76, 147	128, 16, 32, 16	0.5, 0, 0.2, 0.4	1024, 4096	3.8×10^{-4}
ABCE	25, 47, 95, 265	28, 52, 105, 293	128, 32, 32, 16	0.5, 0, 0.2, 0.4	1024, 4096	3.4×10^{-4}
ABDE	25, 39, 129, 229	28, 43, 143, 253	32, 16, 16, 32	0.2, 0.3, 0.3, 0.5	64, 512	1.3×10^{-4}
ACDE	23, 79, 143, 307	25, 87, 158, 340	32, 16, 16, 32	0, 0.2, 0.1, 0.5	128, 4096	1.6×10^{-4}
BCDE	41, 87, 153, 335	45, 96, 169, 371	32, 16, 16, 32	0.5, 0.2, 0.1, 0.5	32, 512	3.9×10^{-4}
ABCDE	17, 35, 87, 121, 215	19, 39, 96, 134, 238	128, 16, 16, 16, 16	0.3, 0.1, 0, 0.1, 0.5	4096, 1024	6.2×10^{-4}

Table S3. Tuned hyperparameters for previous studies regarding multiscale 1D-CNNs with 5-fold cross-validation. In the kernel size combination [21/3, 21/5] [4], the multiscale CNN includes two parallel structures. One structure uses a kernel size of 21 followed by a kernel size of 3, while the other uses a kernel size of 21 followed by a kernel size of 5.

Kernel size combination (pixel interval)	Kernel size combination (cm^{-1})	Channel	Node	Learning rate
[5] [3, 4, 5]	N.A.	32, 32, 32	128	1.0×10^{-4}
[4] [21/3, 21/5]	N.A.	8/32, 16/32	128	1.0×10^{-4}
[6] [3~1009, interval = 10]	N.A.	64 for each kernel size	128	1.0×10^{-5}

Table S4. Tuned hyperparameters for traditional machine learning algorithms with 5-fold cross-validation.

Algorithm	Search space	Best hyperparameter combination
SVM	C: [0.01, 0.1, 1, 10, 100]	10
	kernel: ['poly', 'rbf', 'sigmoid']	rbf
	degree: [1, 2, 3]	1
	gamma: ['scale', 'auto']	scale
LDA	solver: ['svd', 'lsqr', 'eigen']	lsqr
	shrinkage: [None, 'auto', 0.1, 0.5, 0.9]	auto
kNN	n_neighbors: [3, 5, 7, 9]	3
	weights: ['uniform', 'distance']	uniform
	metric: ['euclidean', 'manhattan', 'minkowski']	euclidean

REFERENCES

1. Kučerka, N.; Tristram-Nagle, S.; Nagle, J. F., Structure of Fully Hydrated Fluid Phase Lipid Bilayers with Monounsaturated Chains. *The Journal of Membrane Biology* **2006,** *208* (3), 193-202.
2. Tanaka, K.; Murakami, I.; Mikami, M.; Aoki, D.; Iwamori, M., Reduced expression of gangliosides with GM2-determinant in cervical carcinoma-derived cells after subcutaneous transplantation into nude mice. *Human Cell* **2023,** *36* (3), 1199-1203.
3. Quinn, P. J.; Chapman, D., The dynamics of membrane structure. *CRC Crit Rev Biochem* **1980,** *8* (1), 1-117.
4. Tang, J.-W.; Lyu, J.-W.; Lai, J.-X.; Zhang, X.-D.; Du, Y.-G.; Zhang, X.-Q.; Zhang, Y.-D.; Gu, B.; Zhang, X.; Gu, B.; Wang, L., Determination of Shigella spp. via label-free SERS spectra coupled with deep learning. *Microchemical Journal* **2023,** *189*, 108539.
5. Ding, J.; Lin, Q.; Zhang, J.; Young, G. M.; Jiang, C.; Zhong, Y.; Zhang, J., Rapid identification of pathogens by using surface-enhanced Raman spectroscopy and multi-scale convolutional neural network. *Anal Bioanal Chem* **2021,** *413* (14), 3801-3811.
6. Deng, L.; Zhong, Y.; Wang, M.; Zheng, X.; Zhang, J., Scale-Adaptive Deep Model for Bacterial Raman Spectra Identification. *IEEE J Biomed Health Inform* **2022,** *26* (1), 369-378.
